# Natural killer cells act as an extrinsic barrier for *in vivo* reprogramming

**DOI:** 10.1242/dev.200361

**Published:** 2022-04-22

**Authors:** Elena Melendez, Dafni Chondronasiou, Lluc Mosteiro, Jaime Martínez de Villarreal, Marcos Fernández-Alfara, Cian J. Lynch, Dirk Grimm, Francisco X. Real, José Alcamí, Núria Climent, Federico Pietrocola, Manuel Serrano

**Affiliations:** 1 Institute for Research in Biomedicine (IRB Barcelona), Barcelona Institute of Science and Technology (BIST), Barcelona 08028, Spain; 2 Department of Discovery Oncology, Genentech, South San Francisco, CA 94080, USA; 3 Epithelial Carcinogenesis Group, Spanish National Cancer Research Centre (CNIO), Madrid 28029, Spain; 4 CIBERONC, Madrid 28029, Spain; 5 Department of Infectious Diseases/Virology, Medical Faculty, University of Heidelberg, Heidelberg 69120, Germany; 6 BioQuant, Cluster of Excellence CellNetworks, University of Heidelberg, Heidelberg 69120, Germany; 7 German Center for Infection Research (DZIF) and German Center for Cardiovascular Research (DZHK), partner site Heidelberg, Heidelberg 69120, Germany; 8 Department of Medicine and Life Sciences, Universitat Pompeu Fabra, Barcelona 08003, Spain; 9 HIV Unit, Hospital Clínic - Institut d'Investigacions Biomèdiques August Pi i Sunyer (IDIBAPS), Barcelona 08036, Spain; 10 AIDS Immunopathology Unit, National Center for Microbiology, Institute of Health Carlos III, Majadahonda (Madrid) 28220, Spain; 11 CIBER de Enfermedades Infecciosas, Instituto de Salud Carlos III, Madrid 28029, Spain; 12 Fundació Clínic per a la Recerca Biomèdica (FCRB), Barcelona 08036, Spain; 13 Department of Biosciences and Nutrition, Karolinska Institutet, Huddinge 14152, Sweden; 14 Catalan Institution for Research and Advanced Studies (ICREA), Barcelona 08010, Spain

**Keywords:** Reprogramming, Pluripotency, Plasticity, Immune system, Natural killer cells, NK receptor ligands, Organoids, Mouse

## Abstract

The ectopic expression of the transcription factors OCT4, SOX2, KLF4 and MYC (OSKM) enables reprogramming of differentiated cells into pluripotent embryonic stem cells. Methods based on partial and reversible *in vivo* reprogramming are a promising strategy for tissue regeneration and rejuvenation. However, little is known about the barriers that impair reprogramming in an *in vivo* context. We report that natural killer (NK) cells significantly limit reprogramming, both *in vitro* and *in vivo*. Cells and tissues in the intermediate states of reprogramming upregulate the expression of NK-activating ligands, such as MULT1 and ICAM1. NK cells recognize and kill partially reprogrammed cells in a degranulation-dependent manner. Importantly, *in vivo* partial reprogramming is strongly reduced by adoptive transfer of NK cells, whereas it is significantly increased by their depletion. Notably, in the absence of NK cells, the pancreatic organoids derived from OSKM-expressing mice are remarkably large, suggesting that ablating NK surveillance favours the acquisition of progenitor-like properties. We conclude that NK cells pose an important barrier for *in vivo* reprogramming, and speculate that this concept may apply to other contexts of transient cellular plasticity.

## Abstract (precis)

**Summary:** Cellular reprogramming is inefficient *in vivo* partly because natural killer (NK) cells eliminate cells undergoing reprogramming; transient depletion of NK cells facilitates reprogramming and the emergence of cells with high organoid-formation capacity, which could be important for tissue repair applications.

## INTRODUCTION

The ectopic expression of the four transcription factors OCT4 (encoded by *Pou5f1*), SOX2, KLF4 and MYC (OSKM) enables reprogramming of differentiated cells into induced pluripotent stem cells (iPSCs) (Takahashi and Yamanaka, 2006). Reprogramming *in vivo*, within the context of an adult organism, has also been achieved in mice with inducible transgenic expression of OSKM (Abad et al., 2013; Ohnishi et al., 2014). The process of reprogramming is typically inefficient, both *in vitro* and *in vivo*, and only a minority of cells reach full reprogramming. This is due, at least in part, to the existence of multiple cell-autonomous barriers, such as tumour suppressors, chromatin regulators, transcription factors, signalling pathways and micro RNAs (Haridhasapavalan et al., 2020; Arabacı et al., 2021). In contrast to the extensive knowledge on cell-intrinsic barriers, the extrinsic barriers that limit reprogramming remain largely unknown. In this work, we examine the composition of the immune cell infiltrate during *in vivo* reprogramming and identify a novel role of NK cells as a key barrier for reprogramming.

Transgenic OSKM expression *in vivo* triggers first the loss of cellular identity and formation of dysplastic areas across multiple tissues (Abad et al., 2013; Ohnishi et al., 2014). We refer to this intermediate state as ‘partial’ reprogramming. Interestingly, at this stage, the effects of OSKM are reversible and tissues recover normal histology upon extinction of transgene expression (Ohnishi et al., 2014). Continued expression of OSKM leads to cells expressing markers of embryonic pluripotency, which will ultimately form teratomas in various organs (Abad et al., 2013; Ohnishi et al., 2014). Currently, there is great interest in the potential of partial reprogramming as a strategy for rejuvenation and tissue repair. Ocampo et al. showed that short-term cyclic expression of OSKM improves physiological parameters after injury and extends lifespan in a model of premature aging, with negligible risk of teratoma formation (Ocampo et al., 2016). In line with the pro-regeneration potential of *in vivo* partial reprogramming, transient OSKM (or OSK) expression has been demonstrated to enhance tissue repair in models of muscle injury (Chiche et al., 2017; de Lázaro et al., 2019), skin wound healing (Doeser et al., 2018), optic nerve crush injury, glaucoma and aging-associated loss of vision (Lu et al., 2020) and myocardial infarction (Chen et al., 2021).

The capacity of the adaptive and innate immune cells to recognize and target pluripotent stem cells (PSCs) for elimination has been previously demonstrated. For instance, activated cytotoxic T lymphocytes (CTLs) have the ability to kill PSCs, including multipotent adult germ-line stem cells (maGSCs), embryonic stem cells (ESCs) and iPSCs in a peptide-dependent manner (Dressel et al., 2009). Furthermore, natural killer (NK) cells (Frenzel et al., 2009; Dressel et al., 2010) and the complement system (Koch et al., 2006) play a role in the rejection of stem cells *in vitro* and *in vivo*. NK cells also limit teratoma formation after subcutaneous injection of mouse ESCs, iPSCs and maGSCs (Dressel et al., 2008) or human iPSCs (Benabdallah et al., 2019). The sensitivity of PSCs to NK-mediated clearance has been ascribed to the downregulation of major histocompatibility complex I (MHCI) on the surface of PSCs (a process known as ‘missing self’) and/or to the upregulation of NK-activating ligands (Dressel et al., 2008, 2009, 2010; Frenzel et al., 2009). Although the recognition and elimination of PSCs by the immune system is reasonably well understood, nothing is known about the interplay between the intermediate states of partial reprogramming and the immune system. It is unclear which immune subtypes infiltrate organs undergoing partial reprogramming and the extent to which they influence the outcome of *in vivo* reprogramming.

In the present study, we report that NK cells recognize and kill cells undergoing partial reprogramming. Furthermore, our results suggest that NK cells preferentially eliminate cells endowed with higher plasticity and regenerative capacity.

## RESULTS

### Partial reprogramming elicits immune infiltration in the pancreas

**Fig. 1.**
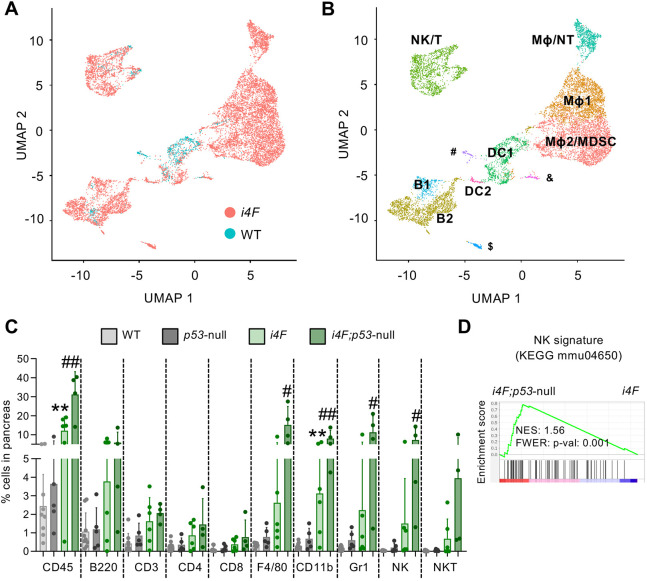
**Immune cell populations infiltrate the pancreas during *in vivo* reprogramming.** (A) UMAP plot visualizing immune cell infiltrates in the pancreas. WT (*n*=2) and *i4F* (*n*=3) mice were treated with doxycycline in the drinking water (1 mg/ml) for 1 week to induce partial reprogramming. (B) UMAP plot of immune cell populations. Clusters were annotated using the most-significant markers of each cluster and the FindAllMarkers function of Seurat (v3). The minor clusters correspond to endothelial cells (#), stellate cells (&) and marginal zone B cells ($). (C) Flow cytometry analysis of the main immune populations infiltrating the pancreas after WT (*n*=9), *p53*-null (*n*=5), *i4F* (*n*=6) and *i4F*;*p53*-null (*n*=4) mice were treated with doxycycline for 7 days. Cells were gated from DAPI^−^/CD45^+^ cells. Data are pooled from two independent experiments and represent mean±s.e.m. **P<0.01 (WT versus *i4F*) and ^#^*P*<0.05, ^##^*P*<0.01 (*i4F* versus *i4F*;*p53*-null) evaluated using the unpaired two-tailed Student's *t*-test. (D) Previously published RNA-seq data generated in our laboratory (Mosteiro et al., 2016) from the pancreas of *i4F* and *i4F*;*p53*-null (high-reprogramming) mice were used to perform gene set enrichment analysis (GSEA) against a published signature (KEGG entry: mmu04650) of NK cell-mediated cytotoxicity. B, B cells; DC, dendritic cells; Mφ, macrophages; MDSC, myeloid derived suppressor cells; NK, natural killer cells; NT, neutrophils; T, T cells.

To interrogate whether the immune system plays a role in *in vivo* reprogramming, we first performed single-cell RNA-sequencing (scRNA-seq) analysis of the pancreata from mice with a doxycycline-inducible transgene expressing the four Yamanaka factors (*i4F*) and from wild-type (WT) mice, all treated with doxycycline for 1 week. Uniform Manifold Approximation and Projection (UMAP) representation showed an abundant infiltration of immune cells in response to reprogramming (Fig. 1A). Cell clusters were annotated according to their most significantly expressed markers (Fig. 1B; Fig. S1). We found that NK cells, T cells, neutrophils (NTs), macrophages (Mφ) and B cells were enriched in pancreata undergoing partial reprogramming (Fig. 1B) and their abundance was further supported by immunohistochemistry analysis (Fig. S2). Of note, the enrichment of NK cells was unique to the pancreas and was not observed in spleen and lymph nodes (Fig. S3). The relative abundance of other immune cell populations tested in spleen and lymph nodes remained unchanged, with the exception of Mφ and NTs, which were enriched in spleen (Fig. S3). We wondered whether the magnitude of immune infiltration in the pancreas would positively correlate with the extent of tissue dysplasia evoked by OSKM expression. As previously reported, *i4F;p53*-null mice are more prone to undergo reprogramming than are *i4F* mice with functional p53 (Mosteiro et al., 2016). We found by flow cytometry a higher proportion of CD45^+^ immune cells in *i4F;p53*-null pancreata compared with their *i4F* counterparts, indicating that the degree of reprogramming correlates with the extent of immune cell infiltration in the pancreas (Fig. 1C). The most abundant cell types infiltrating the reprogramming pancreas belonged to the innate immune system, in particular F4/80^+^ and CD11b^+^ cells (which mostly correspond to Mφ), Gr1^+^ cells (which mostly correspond to NTs and myeloid-derived suppressor cells or MDSCs) and NK cells (Fig. 1C). Of note, bulk transcriptomics analysis performed in the pancreas of *i4F;p53*-null mice revealed a significant enrichment in NK cell cytotoxicity-associated genes [Kyoto Encyclopaedia of Genes and Genomes (KEGG) entry: mmu04650] compared with *i4F* mice (Mosteiro et al., 2016) (Fig. 1D). Based on these observations, we decided to study in detail the role of NK cells during reprogramming as well as exploring the role of Mφ and Gr1^+^ cells.

### NK cells eliminate partially reprogrammed cells *in vitro*

**Fig. 2.**
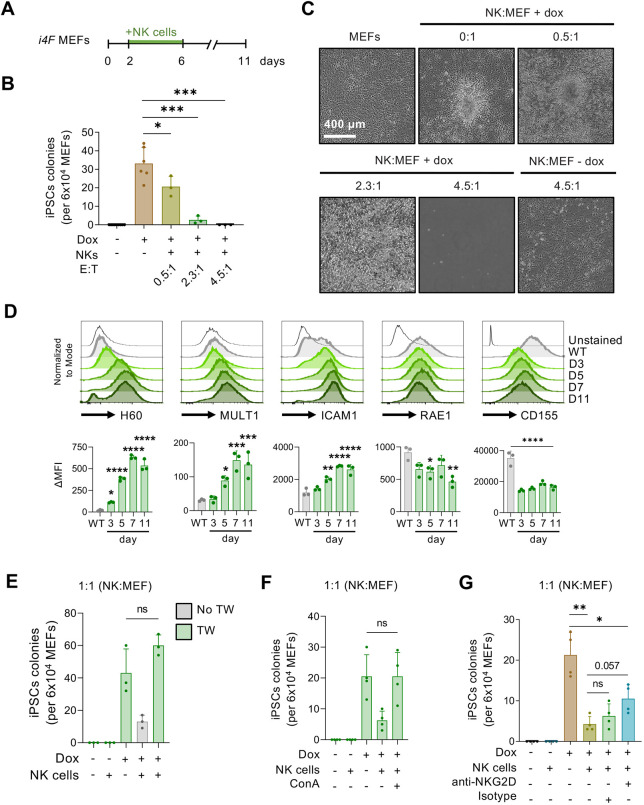
**NK cells eliminate partially reprogrammed cells *in vitro*.** (A) *i4F* MEFs were reprogrammed *in vitro* with doxycycline (Dox; 1 μg/ml) for 11 days. From days 2 to 6, primed NK cells were added to the medium and cells were then co-cultured for 5 days in co-culture medium. On day 11, iPSC colonies were scored by AP staining. (B,C) Quantification (B) and representative images (C) of NK cells co-cultured with *i4F* MEFs with or without doxycycline at E:T (NK:MEF) ratios of 0.5:1, 2.3:1 and 4.5:1. Data pooled from two independent experiments. (D) Delta mean fluorescent intensity (ΔMFI) of the total H60, MULT1, ICAM1, RAE1 and CD155 expression in *i4F* MEFs reprogrammed with doxycycline (1 μg/ml) at different time points (*n*=3). (E) Co-culture experiment in which primed NK cells were seeded in Transwells (TW) at an NK:MEF ratio of 1:1 to avoid cell–cell contact (*n*=3). (F) Co-culture experiment using ConA to disrupt the function of lytic granules secreted by NK cells at an NK:MEF ratio of 1:1 (*n*=4). (G) Co-culture experiment using the blocking antibody anti-NKG2D, which was added to the medium on days 2 and 4 of reprogramming (*n*=4). All data are mean±s.d.; *P<0.05, **P<0.001, ***P<0.001, ****P<0.0001 evaluated using the unpaired two-tailed Student's *t*-test (B,E-G) or one-way ANOVA (D). ns, not significant.

To investigate whether NK cells can directly interfere with the early stages of reprogramming, we performed co-culture experiments. Previous to this, we confirmed that our co-culture medium (containing IL2 and IL15) did not affect the function of NK cells or the reprogramming of fibroblasts, when tested separately (Fig. S4A-C). We then co-cultured primed splenic WT NK cells with *i4F* mouse embryonic fibroblasts (MEFs) at different effector:target (E:T) ratios from day 2 to day 6 of *in vitro* reprogramming, which corresponds to the stage of partial reprogramming. On day 6, NK cells were removed from the culture and reprogramming was continued in standard iPSC medium until formation of iPSC colonies (scored on day 11) (Fig. 2A). Interestingly, we observed that the number of alkaline phosphatase (AP)-positive iPSC colonies was inversely proportional to the number of NK cells in the medium (Fig. 2B,C). At the end of the reprogramming assay (day 11), those plates co-cultured with the highest amount of NK cells had no evidence of surviving fibroblasts, indicating the complete killing of the reprogramming fibroblasts (Fig. 2C). In contrast, non-reprogramming fibroblasts co-cultured with the same amount of NK cells remained viable at the end of the experiment (Fig. 2C). This result indicates that cells undergoing OSKM-induced reprogramming are targeted by NK cells for elimination.

The NK receptors NKG2D [KLRK1; which binds to RAE1, MULT1 (ULBP1) and H60 ligands in mouse cells] (Cerwenka et al., 2000; Carayannopoulos et al., 2002), LFA-1 (ITGAL; which interacts with ICAM1) (Chong et al., 1994) and DNAM-1 [CD226; activated by CD155 (PVR) in mouse] (Stanietsky et al., 2013) have been described as major signalling molecules for NK activation. We studied the expression kinetics of these ligands in reprogramming MEFs by flow cytometry. We observed upregulation of the NKG2D ligands H60 and MULT1 (Fig. 2D). In line with previous observations by flow cytometry (Schwarz et al., 2018), we also detected a progressive upregulation of ICAM1 over the course of reprogramming. We did not observe upregulation of RAE1 and CD155. Taken together, these results show that cells undergoing reprogramming exhibit enhanced expression of a subset of NK stimulatory ligands. We next investigated whether NK-activating ligands are also upregulated during *in vitro* human cell reprogramming using previously published scRNA-seq data from human dermal fibroblasts reprogrammed to naïve iPSCs (Liu et al., 2020). Interestingly, these data showed upregulation of the NK-activating ligand *MICB* together with downregulation of the NK-inhibitory ligands *HLA-A* and *HLA-E* (Fig. S5). These observations suggest that, similar to mouse cells, human cells undergoing reprogramming also become potential targets for NK cells.

To gain mechanistic insights into the role of NK cells during *in vitro* reprogramming, we first asked whether NK cells require physical contact with partially reprogrammed target cells. To address this, we co-cultured primed NK cells with *i4F*-MEFs in Transwell plates. No significant differences were detected in the number of iPSC colonies between *i4F*-MEFs cultured with or without NK cells in Transwells (Fig. 2D). We then tested, in direct co-culture conditions, the involvement of cytotoxic granules in the killing process by using the v-ATPase inhibitor concanamycin A (ConA), which prevents the release of lytic granules by activated NK cells. The presence of ConA abolished the inhibitory action of NK cells on iPSC formation (Fig. 2E). Collectively, these data indicate that partially reprogrammed cells are lysed by NK cells in a process involving direct contact with their target and release of lytic granules.

To evaluate the implication of the NKG2D/ligand interaction in NK cell-mediated recognition and elimination of cells undergoing reprogramming, we co-cultured NK cells and *i4F*-MEFs with an NKG2D-blocking antibody or with the corresponding isotype control. Under this setting, we found that anti-NKG2D treatment partially suppressed the inhibitory effect of NK cells on reprogramming (Fig. 2F). Therefore, these data demonstrate that NK cells target early reprogramming cells via, at least in part, engagement of the NKG2D receptor.

### NK cells activate and degranulate during *in vivo* reprogramming

**Fig. 3.**
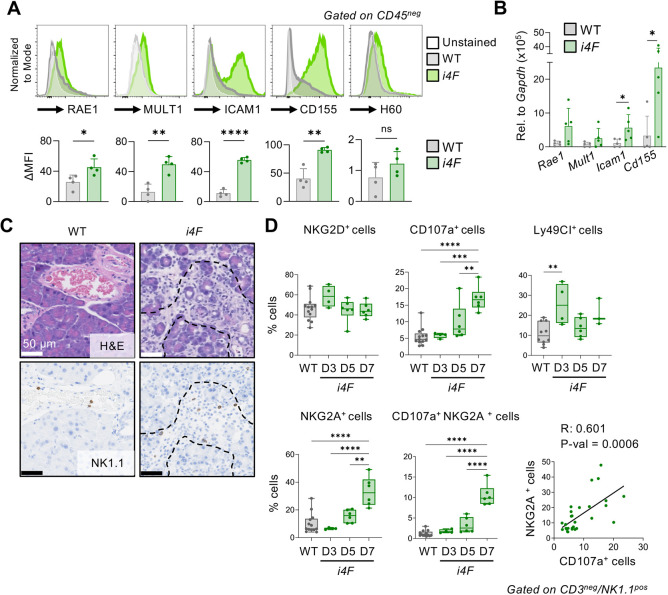
**NK cells recruited to the pancreas of *i4F* mice release lytic granules upon ligand-dependent activation.** (A) Delta mean fluorescent intensity (ΔMFI) of the total RAE1, MULT1, ICAM1, CD155 and RAE1 expression in pancreas of WT and *i4F* mice treated with doxycycline (1 mg/ml) for 7 days (*n*=4). (B) WT and *i4F* mice were treated with doxycycline (1 mg/ml) for 7 days and expression levels of NK-activating ligands (*Rae1*, *Mult1*, *Icam1* and *CD155*) in pancreatic tissue were assessed by RT-qPCR (*n*=5). Data in A,B are mean±s.d. *P<0.05, **P<0.01, ****P<0.0001 evaluated using the unpaired two-tailed Student's *t*-test. (C) Representative H&E (above) and NK1.1 staining (below) of pancreata of WT and *i4F* mice treated with doxycycline for 7 days (*n*=5). (D) Subpopulations of infiltrating NK cells in pancreas undergoing reprogramming on days 3 (D3), 5 (D5) and 7 (D7) of doxycycline treatment. Cell populations were gated on CD3^−^/NK1.1^+^ cells and stained for the indicated activation, degranulation and inhibitory markers. Correlation of NKG2A^+^ and CD107a^+^ cells includes all time points. In the box-and-whisker plots, boxes show median and upper and lower quartiles, and whiskers indicate minimum and maximum values; **P<0.01, ***P<0.001, ****P<0.0001 evaluated using one-way ANOVA (data pooled from four independent experiments).

To dissect the role of NK cells recruited to the pancreas during *in vivo* reprogramming, we analysed the expression of NK-activating ligands in the pancreata of *i4F* mice at an intermediate stage of reprogramming (7 days of doxycycline treatment). In line with our *in vitro* observations, NK-activating ligands were transcriptionally upregulated in the pancreata of *i4F* mice compared with their WT counterparts. By flow cytometry of non-immune cells (CD45^−^ cells), we observed significant upregulation of RAE1, MULT1, ICAM1 and CD155 ligands in pancreas undergoing partial reprogramming (Fig. 3A). In some cases (i.e. *Icam1* and *Cd155*), this was accompanied by a significant increase in mRNA levels (Fig. 3B). We surmise that the different behaviour of CD155 in pancreas versus cultured fibroblasts reflects cell type-specific differences. These results prompted us to examine the localization of infiltrating NK cells in the pancreata of partially reprogrammed mice. Notably, we found NK cells directly surrounding dysplastic cells in pancreas undergoing partial reprogramming (Fig. 3C). In control pancreas, NK cells were only detected within the blood vessels. To further characterize the NK cells infiltrating pancreas during the process of reprogramming, we used cell surface markers to profile the activation status of NK cells at different time points (Fig. 3D). We observed a modest transient cell surface increase in NKG2D on day 3. The transient nature of NKG2D elevation in the cell surface is consistent with the idea that NKG2D engagement by membrane-bound ligands triggers its internalization, which is necessary for intracellular signalling (Coudert et al., 2005; Oppenheim et al., 2005; Wiemann et al., 2005). Interestingly, as early as 5 days after initiating reprogramming, NK cells significantly upregulated the degranulation marker CD107a (also known as LAMP1) (Alter et al., 2004). Finally, we also observed an upregulation of the inhibitory receptors LY49C and LY49I (members of the KLRA family detected together as LY49C/I) and NKG2A (KLRC1), likely reflecting a progressive exhaustion (Ablamunits et al., 2011; Sun et al., 2017). Interestingly, double-positive CD107a^+^ NKG2A^+^ NK cells were progressively upregulated as reprogramming progressed, and these markers were also highly positively correlated when examined separately, suggesting an association between degranulation and inhibition of NK cells. Collectively, these findings indicate that, upon infiltrating the reprogramming pancreas, NK cells manifest hallmarks of activation, including degranulation and upregulation of inhibitory receptors.

### NK cells are a barrier for *in vivo* reprogramming in the pancreas

Based on the above findings, we hypothesized that NK cells might limit *in vivo* reprogramming. We first addressed the question of whether the activity of NK cells was affected by the activation of our transgenic OSKM allele. We confirmed that the OSKM transgene was upregulated in splenic NK cells after 7 days of treatment of *i4F* mice with doxycycline (Fig. S6A). We then isolated NK cells from the spleen of *i4F* and WT mice treated with doxycycline for 5 days *in vivo*. These NK cell isolates were then primed *in vitro* for 6 days with the cytokines IL2 and IL15, while maintaining the presence of doxycycline. Finally, NK activity was measured using YAC-1 cells as target cells, which are sensitive to NK clearance as a result of the lack of MHCI expression (Cikes et al., 1973). The percentage of dead (DAPI^+^) YAC-1 cells did not vary between WT and *i4F* NK cells (Fig. S6B). This result indicates that the expression of our reprogramming OSKM cassette did not significantly alter the activity of NK cells during the time frame of *in vivo* reprogramming studied in the pancreas.

**Fig. 4.**
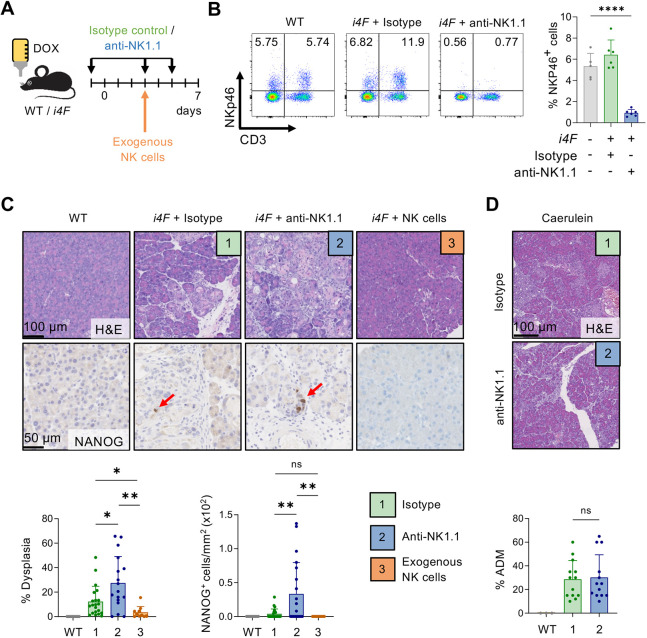
**NK cells are an extrinsic barrier for *in vivo* reprogramming.** (A) WT (*n*=10) and *i4F* mice were treated with doxycycline (Dox; 1 mg/ml) for 7 days. *i4F* mice were treated with either isotype control antibody (1) (*n*=21) or anti-NK1.1 antibody (2) (*n*=17) (days −1, 3 and 5), or received adoptive transfer of 3.8×10^6^ NK cells on day 3 of reprogramming (3) (*n*=10). (B) Representative flow cytometry plots and quantification of NK cells in blood of randomly selected mice on day 5 of reprogramming. NK cells were gated as CD3^−^/NKp46^+^ cells (*n*=5 for WT+dox and *n*=6 for *i4F*+isotype and *i4F*+anti-NK1.1 groups). (C) Representative H&E and NANOG staining of pancreatic tissue (upper) and quantifications of the percentage of dysplasia and number of NANOG^+^ cells (lower). Data pooled from five independent experiments in conditions 1 and 2, and from two independent experiments in condition 3. (D) Representative H&E images of caerulein-induced pancreatitis (100 mg/kg, 7 times/day) treated for 2 days with either isotype control or anti-NK1.1 (*n*=13) (upper). Quantification of the percentage of acinar-to-ductal metaplasia (ADM) in the pancreata. All data are mean±s.d.; *P<0.05, **P<0.01, ****P<0.0001 evaluated using the unpaired two-tailed Student's *t*-test. ns, not significant.

Having established that NK activity was not significantly affected by OSKM expression in our mouse model, we then addressed the effect of eliminating NK cells on partial reprogramming. To do so, we depleted NK cells using a monoclonal antibody against NK1.1 (KLRB1C), and NK depletion was confirmed by flow cytometry using anti-NKp46 (NCR1) (Fig. 4A,B). We noted that NK1.1-depleted reprogrammed mice suffered more profound weight loss compared with reprogrammed mice treated with isotype control (Fig. S7A). Moreover, serum amylase levels, which increased proportionally to the levels of dysplasia in our mouse model (Fig. S7B), were significantly upregulated in anti-NK1.1-treated *i4F* mice compared with isotype control-treated *i4F* mice (Fig. S7C). Histologically, the absence of NK1.1^+^ cells resulted in larger areas of pancreatic dysplasia and a higher number of NANOG^+^ cells, a marker of the late stages of reprogramming (Fig. 4C). Based on these results, we propose that NK cells hinder the process of *in vivo* reprogramming by eliminating emerging partially reprogrammed cells. To further strengthen this hypothesis, we tested whether the adoptive transfer of exogenous NK cells reduced the normal levels of reprogramming in the pancreas. Thus, NK cells from WT donor mice were injected on day 3 of reprogramming. Importantly, exogenous transfer of NK cells dramatically reduced the efficiency of reprogramming, and no NANOG^+^ cells were detected (Fig. 4C).

We also examined whether NK cells have a role in other models of tissue plasticity in the pancreas. In particular, we focused on caerulein treatment, which is known to induce acinar-to-ductal metaplasia (ADM), a histological lesion that also implies reversible changes in cell identity (Shibata et al., 2018). Importantly, NK1.1^+^ cell ablation did not alter the amount of caerulein-induced ADM (Fig. 4D). These results reinforce the concept that NK cells are particularly relevant during *in vivo* partial reprogramming.

**Fig. 5.**
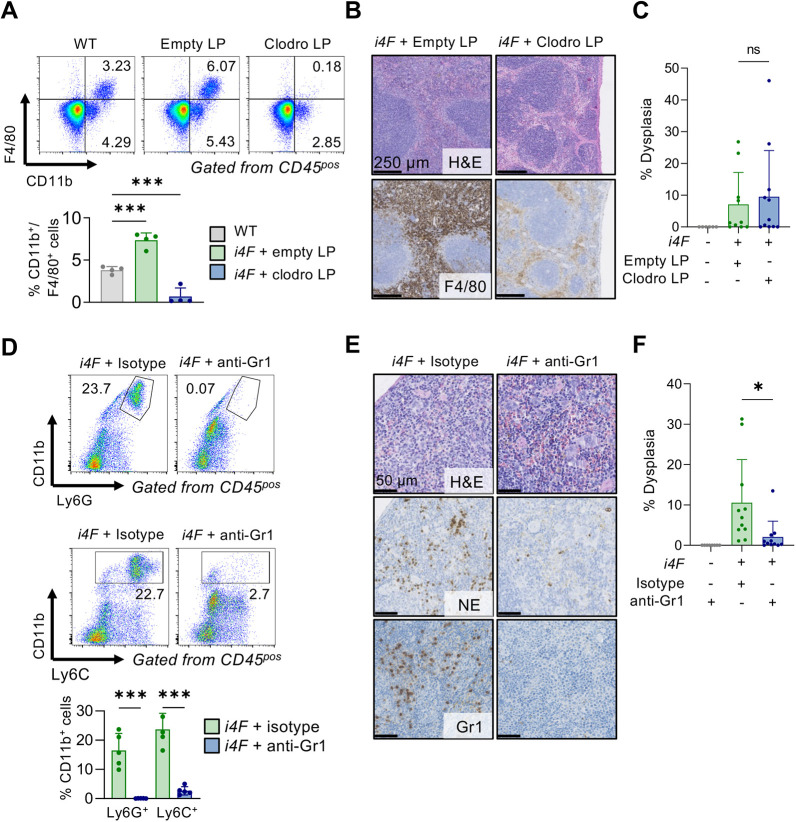
**Mφ and Gr1^+^ cell depletion during *in vivo* reprogramming.** (A) Representative flow cytometry plots and quantification of F4/80 and CD11b double-positive populations in blood 16 h after liposome (LP) were administered intraperitoneally on day 3 of reprogramming. Mice were randomly selected (*n*=4). Cells were gated from CD45^+^ cells. (B) Representative H&E and F4/80 immunohistochemistry of the spleen of mice treated with doxycycline and empty or clodronate LP for 7 days. (C) Dysplasia quantification in the pancreas of mice reprogrammed with empty or clodronate LP for 7 days (*n*=6 for WT, *n*=10 for empty LP and *n*=11 for clodronate LP groups). (D) Representative flow cytometry plots and quantification of Ly6C and CD11b, and Ly6G and CD11b populations in blood on day 5 from reprogramming from mice treated with anti-*Gr1* or isotype control antibodies. Cells were gated from CD45^+^ cells. (E) Representative H&E, neutrophil elastase (NE) and *Gr1* immunohistochemistry in the spleen of partially reprogrammed mice treated with anti-*Gr1* or isotype control antibodies for 7 days. (F) Dysplasia quantification in the pancreas of mice reprogrammed for 7 days with either anti-*Gr1* or isotype control (*n*=8 for WT and *n*=11 for *i4F* and anti-*Gr1* groups). All data are mean±s.d.; *P<0.05, ***P<0.001 evaluated using the unpaired two-tailed Student's *t*-test. ns, not significant.

Finally, we focused on two other immunological cell populations that also infiltrate the pancreas during reprogramming, namely, Mφ and Gr1^+^ cells, the latter including NTs and MDSCs (Fig. 1C). Clodronate-mediated depletion of phagocytic cells (Fig. 5A,B) failed to modify the reprogramming process significantly (Fig. 5C). To deplete Gr1^+^ cells, we used an anti-Gr1 antibody (clone RB6-8C5) that recognizes the antigenic markers Ly6G and Ly6C, mostly expressed in NTs and MDSCs (Fig. 5D,E). Interestingly, anti-Gr1 treatment strongly reduced reprogramming in the pancreas (Fig. 5D). Considering that NTs and MDSCs suppress the activity of NK cells in different contexts, such as cancer (Bruno et al., 2019; Li et al., 2020; Sun et al., 2020; Zalfa and Paust, 2021), these results are consistent with a scenario in which NK cells are negatively regulated by infiltrating Gr1^+^ cells.

### NK cells are a barrier for *in vivo* reprogramming in the liver

**Fig. 6.**
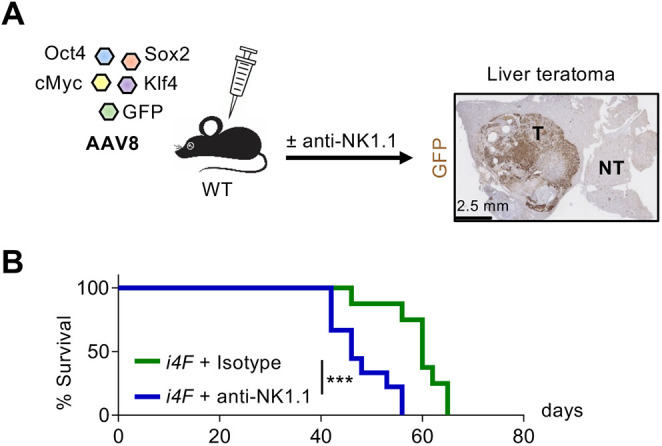
**NK1.1^+^ cell depletion enables full reprogramming and promotes teratoma formation.** (A) WT mice were retro-orbitally injected with scAAV8 SFFV-hCO-O/K/S/M. A scAAV8 vector encoding GFP was additionally added as tracer. Anti-NK1.1 or isotype control antibodies were injected intraperitoneally on days −1, 3 and 5 during the first week, and then once a week until liver teratomas were palpable. (B) Survival curve upon teratoma formation in the liver. Data pooled from two independent experiments (*n*=9). Data are percentage of alive mice; ***P<0.001 evaluated using log-rank (Mantel-Cox) test. NT, nonteratoma; T, teratoma.

To further test the role of NK cells during *in vivo* reprogramming, we shifted to a different experimental model previously reported by us, based on the delivery of the four Yamanaka factors using adeno-associated viruses (AAVs) (Senís et al., 2018). WT mice were inoculated with a mixture of AAV8 viruses, which exhibit high tropism for the liver (Zincarelli et al., 2008), carrying each of the four Yamanaka factors and GFP. As a readout and for simplicity, rather than testing partial reprogramming, we allowed the process to continue until full reprogramming and measured the formation of teratomas (Fig. 6A). Importantly, NK1.1-depleted mice developed teratomas earlier compared with mice with NK cells, further supporting the negative role of NK cells during *in vivo* reprogramming (Fig. 6B). These observations are compatible with the idea that NK cells act as a general barrier for reprogramming in the liver, similar to what we have reported in the pancreas. However, in this case, we cannot discriminate whether NK cells are acting at the partial reprogramming stage and/or at the later stages of reprogramming and teratoma formation.

### Depletion of NK1.1^+^ cells allows the survival of highly plastic pancreatic cells

**Fig. 7.**
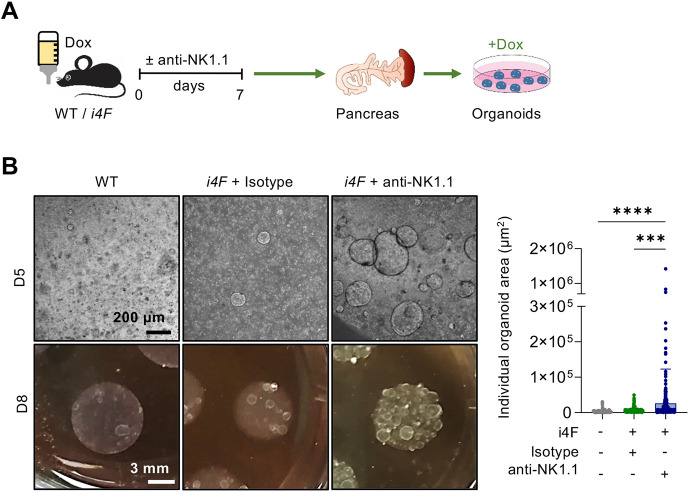
**NK1.1^+^ cell depletion promotes the survival of pancreatic cells with high plasticity.** (A) Mice were treated with doxycycline (Dox) and anti-NK1.1 or isotype control antibodies for 7 days. Pancreata were dissociated to the single cell level and 3×10^5^ cells/well per condition were embedded in Matrigel (*n*=3) (B). Representative images on day 5 (D5) and day 8 (D8) after seeding (left), and quantification of organoids size at D10 (right) (each dot represents one organoid from three biological replicates; ten images were measured per sample). Data are mean±s.d.; ***P<0.001, ****P<0.0001 evaluated using the unpaired two-tailed Student's *t*-test.

To further investigate the role of NK cells as a barrier for reprogramming, we used an *ex vivo* system based on pancreatic organoids as a proxy to monitor the abundance of cells with stem cell-like properties (Clevers, 2016). We reprogrammed mice for 7 days, while treating them with isotype control or anti-NK1.1^+^ antibodies (Fig. 7A). We then prepared single cell pancreatic extracts, and an equal number of cells per condition were embedded in Matrigel in the continuous presence of doxycycline. All organoids grew as homogeneous, hollow spheres composed of a single-layer epithelium, as previously described (Huch et al., 2013). Interestingly, on day 5 post-embedding, organoids originating from anti-NK1.1-treated animals were significantly larger than those derived from *i4F* mice treated with isotype control and, on day 8, the size difference was evident even macroscopically (Fig. 7B). This observation demonstrates that NK cells eliminate partially reprogrammed cells endowed with high plasticity and the capacity to form organoids.

## DISCUSSION

In this study, we provide direct evidence that the host immune system and, specifically NK cells, targets partially reprogrammed cells during the first stages of OSKM expression. We show that NK depletion using antibodies significantly improves the efficiency of *in vivo* reprogramming, in both the pancreas and the liver. Conversely, when exogenous NK cells are adoptively transferred to an *i4F* mouse host, dysplastic areas in the pancreas, indicative of partial reprogramming, are barely detectable. These data, together with the infiltration of NK cells in dysplastic tissue, support the notion that NK cells eliminate cells undergoing reprogramming.

Our data also provide insights into the mechanisms by which NK cells target reprogrammed cells. We show *in vivo* and *in vitro* that partially reprogrammed cells upregulate NK-activating ligands. In particular, we observed *in vivo* the upregulation of MULT1, ICAM1 and CD155, which bind and activate the NK receptors NKG2D, LFA1, and DNAM1, respectively. All these receptors are known to stimulate NK killing activity toward target cells expressing their cognate ligands (Chong et al., 1994; Cerwenka et al., 2000; Carayannopoulos et al., 2002; Stanietsky et al., 2013). The NKG2D receptor is one of the best-known stimulating receptors and is relevant in multiple processes involving NK cells, including autoimmune diseases, viral and bacterial infections, and rejection of transplants (Siemaszko et al., 2021). In our *in vitro* co-culture system, blocking anti-NKG2D antibodies partially rescued the formation of iPSC colonies despite the presence of NK cells. We conclude that NKG2D binding is an important process for the activation of NK cells during partial reprogramming. Our results do not exclude the potential contribution of other stimulating receptors, such as LFA1 and DNAM1, to the negative effect of NK cells on reprogramming.

During *in vivo* reprogramming, the protein levels of NKG2D detected by cytometry modestly increased during the early reprogramming phase, although they did not reach statistical significance and rapidly returned to normal levels. This is in line with the concept that NKG2D engagement leads to its endocytosis, and this is important for the appropriate activation of NK cells (Coudert et al., 2005; Oppenheim et al., 2005; Wiemann et al., 2005). We also observed an increase in the cell surface expression of the NK-inhibitory receptor NKG2A. This inhibitory receptor has been associated with NK cell exhaustion in humans (Ablamunits et al., 2011; Sun et al., 2017). Parallel to NKG2A, we also observed a progressive upregulation of the degranulation marker CD107a. Collectively, these findings indicate that NK cells infiltrate partially reprogrammed pancreata and, upon exposure to NK-activating ligands, release lytic granules and upregulate inhibitory receptors, possibly as part of the acquisition of an exhausted phenotype.

Our cytometry data indicated that multiple immune populations, notably Mφ, MDSCs and NTs, accumulate in the pancreas upon *in vivo* reprogramming. No significant changes were detected in reprogramming efficiency when Mφ were depleted using clodronate liposomes. We surmise that this may reflect the presence of Mφ subpopulations with opposing effects on reprogramming, which is in line with the high heterogeneity and plasticity of Mφ. In contrast, depletion of MDSCs and NTs significantly reduced *in vivo* reprogramming in the pancreas. It has been extensively described that MDSCs suppress NK functions in different contexts, such as hepatitis C virus (HCV) infection (Goh et al., 2016), hepatocellular carcinoma (HCC) (Hoechst et al., 2009) and other types of solid and haematological malignancies (Tumino et al., 2021; Zalfa and Paust, 2021). NTs also impair the cytotoxicity and infiltration capability of NK cells in cancer (Spiegel et al., 2016; Li et al., 2020; Sun et al., 2020). Therefore, our data are compatible with a model in which Gr1^+^ cells counteract the cytotoxic activities of NK cells against partially reprogrammed cells.

We wondered whether depletion of NK cells during OSKM expression could facilitate the emergence of cells with progenitor features. To explore this, we examined the capacity of partially reprogrammed pancreatic cells to form organoids. Interestingly, pancreatic organoids derived from NK-depleted mice grew significantly more compared with organoids derived from mice treated with an isotype control. This suggests that the absence of NK cells facilitates the formation of progenitor-like cells *in vivo* upon OSKM expression. In the future, it will be relevant to explore whether organoids from NK-depleted partially reprogrammed tissues display better engraftment properties and improve tissue regeneration compared with organoids from tissues reprogrammed under the surveillance of NK cells.

Cellular dedifferentiation is emerging as a general process during tissue repair and regeneration (Yao and Wang, 2020). The insights obtained in our study, which also involve *in vivo* dedifferentiation, could be applicable to pathophysiological settings of tissue repair that involve NK cells.

## MATERIALS AND METHODS

### Mice

All mice were bred and maintained at the animal facilities of the Barcelona Science Park in strict accordance with Spanish and European Union regulations. Animal procedures were performed according to protocols approved by the Animal Care and Use Ethical Committee (IACUC) of Barcelona Science Park and the Catalan Government. Mice were housed in a specific pathogen-free (SPF) barrier area with access to *ad libitum* standard chow diet. Animal experiments were designed and conducted with consideration of the ARRIVE guidelines and mice were sacrificed when they presented signs of morbidity, in accordance with the Guidelines for Humane Endpoint for Animals Used in Biomedical Research from the Council for International Organization of Medical Sciences (CIOMS). Reprogrammable *i4F* mice were previously generated in the laboratory on a pure C57BL/6J.Ola.Hsd genetic background (Abad et al., 2013). The *i4F-B* strain contains a ubiquitous doxycycline-inducible transgene encoding OSKM inserted in the *Pparg* gene and the reverse tetracycline-controlled transactivator (rtTA) within the *Rosa26* locus. All mice were heterozygous for both OSKM and rtTA transgenes. All experiments were performed with male and female mice of 8-16 weeks of age.

### Animal procedures

Doxycycline hyclate BioChemica (PanReac, 234-198-7, A2951) was administered in the drinking water (1 mg/ml) supplemented with 7.5% sucrose for 7 days. Immune populations were depleted by intraperitoneal injection of 200 μg of anti-NK1.1 (Bio X Cell, clone PK136) or 200 μg of anti-Gr1 (Bio X Cell, clone RB6-8C5) on days −1, 3 and 5 of reprogramming. Isotype controls IgG2a (Bio X Cell, clone C1.18.4) and IgG2b (Bio X Cell, clone LTF-2) were used in control groups, respectively. For the depletion of Mφ, mice were treated with 200 μl of either clodronate or empty liposomes (Liposoma BV, CP-010-010) retro-orbitally on days 1, 3 and 6 of reprogramming. Mild acute pancreatitis was induced by seven intraperitoneal injections, given once per hour, of a pancreatic secretagogue cholecystokinin (CCK) analogue called caerulein (Bachem, 40304510001) at 100 μg/kg for 2 consecutive days. A second group of control animals received injections of PBS only. Mice were sacrificed on day 4 after the first injection. All animals were sacrificed by cervical dislocation, unless blood extraction from the heart was performed. In that case, mice were sacrificed in a CO_2_ chamber.

### NK cell isolation and adoptive transfer

NK cells were enriched from the spleen of WT C57BL/6J mice via negative selection (Miltenyi Biotec NK cell isolation kit, 130-115-818) and treated with 50 U/ml IL2 (PeproTech, 212-12) and 50 ng/ml IL15 (PeproTech, 210-15) for 24 h in 96 well-plates with U-shaped bottoms (1×10^5^ NK cells/well). NK cell culture medium comprised RPMI-1640 (Sigma-Aldrich, R8758) supplemented with 10% fetal bovine serum (FBS) (10270106), 2 mM l-glutamine (25030024), 100 μg/ml penicillin-streptomycin (15070063), 10 mM HEPES (15630056), non-essential amino acids (11140035, all from Life Technologies), and 0.5 mM sodium pyruvate (Gibco, 11360-070). The following day (day 3 of reprogramming), 3.8×10^6^ NK cells were injected retro-orbitally into recipient mice in 150 μl PBS.

### scAAV8 injections

AAV8 virus was kindly provided by Dr Dirk Grimm (Heidelberg University Hospital, Germany) and was produced as previously described (Senís et al., 2018). WT male mice were retro-orbitally injected with scAAV8 SFFV-hCO-O/K/S/M vectors at doses of 1×10^11^ vg/vector. A scAAV8 vector encoding GFP was added as a tracer at the same concentrations. Throughout the experiment, 200 μg of anti-NK1.1 (clone PK136) or its isotype control, IgG2a (clone C1.18.4), were injected intraperitoneally on days −1, 3 and 5 during the first week, and then once a week until the end of the experiment. Mice were sacrificed when teratomas were palpable.

### Single cell preprocessing and analysis

To isolate all pancreatic cell types, tissue was digested using 1 mg/ml collagenase P (Sigma-Aldrich, 11213865001), 2 U/ml dispase II (Life Technologies, 17105041), 0.1 mg/ml soybean trypsin inhibitor (Life Technologies, 17075-029) and 0.1 mg/ml DNase I (Sigma-Aldrich, D4513) in HBSS with Ca^2+/^Mg^2+^ (Life Technologies, 14025050). Tissue dissociation was performed using the gentle MACS™ Octo Dissociator (Miltenyi Biotech) at 37°C for 40 min. The tissue was further digested with 0.05% trypsin-EDTA (Life Technologies, 25300062) for 5 min at 37°C and erythrocytes were removed with red blood cell lysis buffer (BioLegend, 420301). Cells were loaded onto a 10x Chromium Single Cell Controller chip B (10x Genomics) as described in the manufacturer's protocol (Chromium Single Cell 3′ GEM, Library & Gel Bead Kit v3, PN-1000075). Generation of gel beads in emulsion (GEMs), barcoding, GEM-RT clean up, cDNA amplification and library construction were performed following the manufacturer's recommendations. Libraries were loaded at a concentration of 1.8 pM and sequenced in an asymmetrical pair-end format in a NextSeq500 instrument (Illumina). For data analysis, chromium 10x v3 sequencing files were demultiplexed, aligned to the reference genome [GRCm38 (mm10)] and counts were generated using Cell Ranger software. Barcodes, features and count matrices were loaded into Seurat (v3) for downstream analysis. Each condition (WT and *i4F*) was analysed separately as a merge from the corresponding replicates. Ambient RNA contamination was estimated and corrected using SoupX (Young and Behjati, 2020). Cells expressing less than 150 genes and more than 5% of mitochondrial genes were excluded. Dimensionality of the datasets was set to the first 20 principal components based on the result of the JackStraw analysis and the stabilization of the elbow plot. Normalization was carried out using the SCTranform function (Hafemeister and Satija, 2019). Cluster stability was visualized at different resolutions using clustree (Zappia and Oshlack, 2018). Resolution was set to 0.2 for the two datasets (WT and *i4F*). Clusters were manually annotated based on the significant markers using the FindAllMarkers function. Immune cells for each dataset were extracted as raw counts and merged for the immune combined analysis. Normalization, dimensionality reduction, clustering and cell annotation were carried out in the immune dataset as mentioned above. All datasets were visualized using UMAP projection (Becht et al., 2019).

### Amylase analysis

Serum was obtained from WT (*n*=6) and *i4F* mice treated with isotype control (*n*=6) or anti-NK1.1 antibodies (*n*=6) on day 7 of doxycycline (1 mg/ml) administration in water. Samples were analysed with an amylase kit (Spinreact, SP41201) in a Spinlab 100 (Spinreact, 9-9059) machine.

### Cell culture

Primary MEFs were obtained from *i4F* embryos at embryonic day (E) 13.5 as previously described (Serrano et al., 1997). MEFs were maintained in Dulbecco's modified Eagle's medium (DMEM) (Life Technologies, 10569010) supplemented with 10% heat-inactivated FBS (Life Technologies, 10270106) and penicillin-streptomycin (Life Technologies, 15070063). At passage 1, 6×10^4^ MEFs were seeded in 12 well-plates (Corning, 353043) or 3×10^6^ MEFs were seeded in six-well plates (Corning, 353046), according to the experiment, and cultured with iPSC medium, comprising high-glucose DMEM (Life Technologies, 10569010) supplemented with 15% KnockOut Serum Replacement (KSR) (Life Technologies, 10828028), leukaemia inhibitory factor (LIF) (1000 U/ml; Merck Chemicals, ESG1107), nonessential amino acids (Life Technologies, 11140035), penicillin-streptomycin and 100 μM β-mercaptoethanol (Life Technologies, 31350010). Cells were treated with doxycycline (Sigma-Aldrich, A2951.0025) at 1 μg/ml to induce OSKM-cassette expression. Medium was changed every other day until iPSC colonies appeared on day 11. Cells were then fixed with 4% paraformaldehyde (Aname SL, 15710), washed with PBS and incubated from 30 min to 1 h in AP Blue Membrane Substrate Solution (Sigma-Aldrich, AB0300). AP^+^ colonies per well were scored to determine the efficiency of reprogramming. Cells were maintained in a humidified incubator at 37°C with 5% CO_2_. Cultures were routinely tested for mycoplasma and were always negative.

### Co-culture experiments

For co-culture experiments, NK cells were isolated and activated *in vitro* overnight as described above. NK cells were co-cultured with MEFs from day 2 to day 6 of *in vitro* reprogramming in co-culture medium comprising DMEM (containing l-glutamine and sodium pyruvate) (Life Technologies, 10569010) supplemented with 10% FBS (Life Technologies, 10270106), LIF (1000 U/ml), nonessential amino acids, 10 mM HEPES, penicillin-streptomycin, 100 μM β-mercaptoethanol, 50 U/ml IL2, 50 ng/ml IL15 and doxycycline (1 μg/ml). On day 6, NKs were removed and the culture was washed with PBS once before adding iPSC medium with doxycycline. *In vitro* reprogramming continued until the appearance of iPSC colonies around day 11. Conditions with different E:T ratios (NK:MEF) were used in the same experimental setup (0.5:1, 2.3:1 and 4.5:1). Reprogrammed MEFs non-co-cultured with NK cells were also cultured in the presence of co-culture medium from day 2 to day 6. To test the kill mechanism, NK:MEF co-culture was performed in the presence of the granule exocytosis inhibitor ConA (100 nM) (Life Science, 27689) at an E:T ratio of 1:1.

For the Transwell experiment, NK cells were seeded on top of 12 mm Transwell with 0.4 µm Pore Polycarbonate Membrane Insert (Corning, 3401) on day 2 of reprogramming (E:T ratio 1:1). Transwells were removed on day 6 of reprogramming. NKG2D receptors were blocked by adding mouse NKG2D/CD314 antibody (20 μg/ml) (Bio-Techne R&D Systems, MAB1547-500) to the co-culture on days 2 and 4 of reprogramming (E:T ratio 1:1). All co-culture experiments were performed in 12-well plates (Corning, 353043). For cytotoxic assays, YAC-1 cells were kindly donated by Dr Domingo Barber [National Center for Biotechnology-Spanish National Research Council (CNB-CSIC), Madrid, Spain]. NK cells were isolated from *i4F* and WT mice treated with doxycycline for 5 days. They were primed *in vitro* with IL2 and IL15 in the presence of doxycycline (1 μg/ml) for 6 days, and then co-cultured with YAC-1 cells for 4 h. DAPI^+^ YAC-1 cells were quantified by flow cytometry.

### Pancreatic organoids

Each pancreas was dissociated to the single cell level as described above. For all experimental conditions, the same number of cells (3×10^5^) were embedded in 200 μl growth factor-reduced Matrigel drops (Corning, 356231) and cultured in pancreatic organoid medium, as described previously (Huch et al., 2013). Medium was refreshed every 2-3 days and was supplemented with doxycycline (1 μg/ml) to activate the OSKM cassette. On day 5 after seeding, ten representative 4× fields per sample were taken using ImageJ software to measure the organoid size. All analyses were conducted blind.

### Reverse transcription-PCR

For pancreas samples, total RNA was isolated by acid guanidinium thiocyanate-phenol-chloroform extraction. Up to 5 μg of total RNA was reverse transcribed into cDNA using an iScript Advanced cDNA Synthesis Kit (Bio-Rad, 172-5038). Quantitative real time-(RT-q)PCR was performed using Sybr Green Power PCR Master Mix (Promega Biotech, A6002) in a QuantStudio 6 Flex thermocycler (Applied Biosystems). *Gapdh* served as an endogenous normalization control. The primers used are listed in Table S1.

### Immunohistochemistry of tissue samples

Samples were fixed overnight at 4°C with neutral buffered formalin (Sigma-Aldrich, HT501128-4L). Paraffin-embedded tissue sections (2-3 μm) were dried at 60°C overnight and dewaxed. For Haematoxylin & Eosin (H&E) staining, a standard protocol using CoverStainer (Dako-Agilent) was performed. For some antibodies, immunohistochemistry was performed using a Ventana discovery XT (Roche) for 60 min: Nanog D2A3 (Cell Signaling Technology, 8822) at 1:100-1:250; F4/80 (eBioscience, clone BM, 14-4801-85) at 1:100; and CD45R/B220 (BD Biosciences, 550286) at 1:200. For the remaining antibodies, immunohistochemistry was performed manually or with the Leica Bond RX platform: CD3 (Dako-Agilent, 11503) at 1:10 for 120 min; CD4 (Sino Biological, 50134R001) at 1:2000 for 120 min; CD8α [EPR20305] (Abcam, ab209775) at 1:1000 for 120 min; Klrb1c/CD161c (E6Y9G) (encoding NK1.1 antigen) (Cell Signaling, 39197S) at 1:500 for 120 min; NE (Abcam, ab68672) at 1:1000 for 120 min; and FoxP3 (Cell Signaling, 12653) at 1:750 for 60 min. Antigen retrieval for Nanog and CD45R/B220 was performed with Cell Conditioning 1 (CC1) buffer (Roche, 950-124) and for F4/80 with proteinase K (Dako-Agilent, S3020) for 5 min at room temperature (RT). The secondary antibodies used were OmniMap anti-Rat HRP (Roche, 760-4457, ready to use) or OmniMap anti-Rb HRP (Roche, 760-4311, ready to use). Blocking was performed with casein (Roche, 760-219). Antigen–antibody complexes were revealed with ChromoMap DAB Kit (Roche, 760-159). For CD3, CD4, CD8α, NE, FoxP3 and Klrb1c/CD161c, antigen retrieval was performed with BOND Epitope Retrieval Solution 2 (Leica Biosystems, AR9640), Envision FLEX Target retrieval Solution HIGH pH (Dako-Agilent, K8004) or Trizma Base/EDTA pH 9.0 (T6066-1 Kg, E51345006). Blocking was performed with Peroxidase-Blocking Solution at RT (Dako-Agilent, S2023) and 5% goat normal serum (Life Technology, 16210064) mixed with 2.5% BSA diluted in wash buffer for 10 min and 60 min at RT. The secondary antibody used was BrightVision poly HRP-anti-rabbit IgG (VWR International, ready to use), incubated for 45 min (ImmunoLogic, DPVR-110HRP) or polyclonal goat anti-mouse (Dako-Agilent, P0447) at 1:100 for 30 min. Antigen–antibody complexes were revealed with 3-3′-diaminobenzidine (Leica Biosystems, RE7230-K). Sections were counterstained with Haematoxylin (Dako-Agilent, CS700) and mounted with Mounting Medium, Toluene-Free (Dako-Agilent, CS705) using a Dako CoverStainer. Specificity of staining was confirmed by staining with rat IgG (R&D Systems, 6-001-F), mouse IgG (Abcam, ab37355) or a rabbit IgG (Abcam, ab27478) isotype controls. Brightfield images were acquired with a NanoZoomer-2.0 HT C9600 digital scanner (Hamamatsu) equipped with a 20× objective and visualized at 1.8 gamma correction.

### Image analysis

Brightfield images of immunohistochemistry were quantified in a blinded way using QuPath software 0.1.2 (Bankhead et al., 2017) with standard DAB detection methods. Reprogramming efficiency was evaluated and quantified by histopathological assessment of dysplastic areas in the pancreas.

### Flow cytometry

Blood to assess immune cell depletion was collected in Microvette EDTA-coated tubes (Laborimpex, 16.444) and incubated with red blood cell lysis buffer (BioLegend, 420301) for 5 min. Cells were then washed once with PBS and pre-incubated for 5 min with Mouse BD Fc Block containing purified anti-mouse CD16/CD32 mAb 2.4G2 at 1 μg/1 million cells in 100 μl (BD Biosciences, 553142) of FACS buffer (0.5% BSA and 5 mM EDTA in PBS) for 15 min at 4°C. After a washing step with FACS buffer, cells were incubated with the appropriate antibody for 40 min at 4°C (Table S2). For analysis of infiltrating immune cell populations, pancreata were digested as described above and immune cell types were stained following the same protocol (Table S2), but keeping the samples at RT. Spleen and inguinal lymph nodes were mechanically processed into single cell suspensions. Erythrocytes were lysed using isotonic ammonium chloride solution before performing antibody staining. Single cell suspensions were subsequently incubated with TruStain FcX (anti-mouse CD16/32) antibody (1:100; BioLegend, 101320) for 10 min on ice. After a washing step with FACS buffer, cells were incubated with the appropriate antibody for 20 min at 4°C (Table S2). After three washes with FACS buffer, DAPI Fixable Violet (Life Technologies, D1306) was used to exclude dead cells. FACS analyses were performed using a Gallios Flow Cytometry System (Beckman Coulter) or a FACSAria Fusion (BD Biosciences) with BD FACSDiva software (v.8.0.1), depending on the experiment.

For the characterization of NK cell surface receptors, pancreatic cells were stained with the LIVE/DEAD™ Fixable Blue Dead Cell Stain Kit (Thermo Fisher Scientific, L23105) for 15 min to evaluate viability. After a washing step with PBS, cells were incubated for 20 min with a combination of anti-mouse antibodies (Table S2). Isotype control antibodies were used as negative controls. Cells were analysed with a BD LSRFortessa™ Flow Cytometer. All data were analysed by FlowJo v10 software (BD Biosciences) and GraphPad Prism v9.0.1.

### Statistical analysis

The data were analysed using GraphPad Prism v.9.0.1 software and represented as mean±s.d. or mean±s.e.m. of independent biological replicates (mice or clones of MEFs). Statistical analyses were performed using an unpaired two-tailed Student's *t*-test to compare the means between two different groups. Means of multiple groups were compared by one-way analysis of variance (ANOVA). Differences were considered significant based on the *P*-value (*P<0.05; **P<0.01; ***P<0.001; ****P<0.0001, unless otherwise stated). Gene set enrichment analyses (GSEAs) were performed using data from Mosteiro et al. (2016) and Kyoto Encyclopaedia of Genes and Genomes (KEGG) entry mmu04650 (https://www.genome.jp).

## Supplementary Material

Supplementary information

Reviewer comments
