# Supplementary material for: Natural killer cells act as an extrinsic barrier for in vivo reprogramming
Source: Development. 2022 Apr 22;149(8):dev200361. doi: 10.1242/dev.200361 (PMC9124575; doi:10.1242/dev.200361)
Supplement: Supplementary information [file develop-149-200361-s1.pdf]

Figure S1

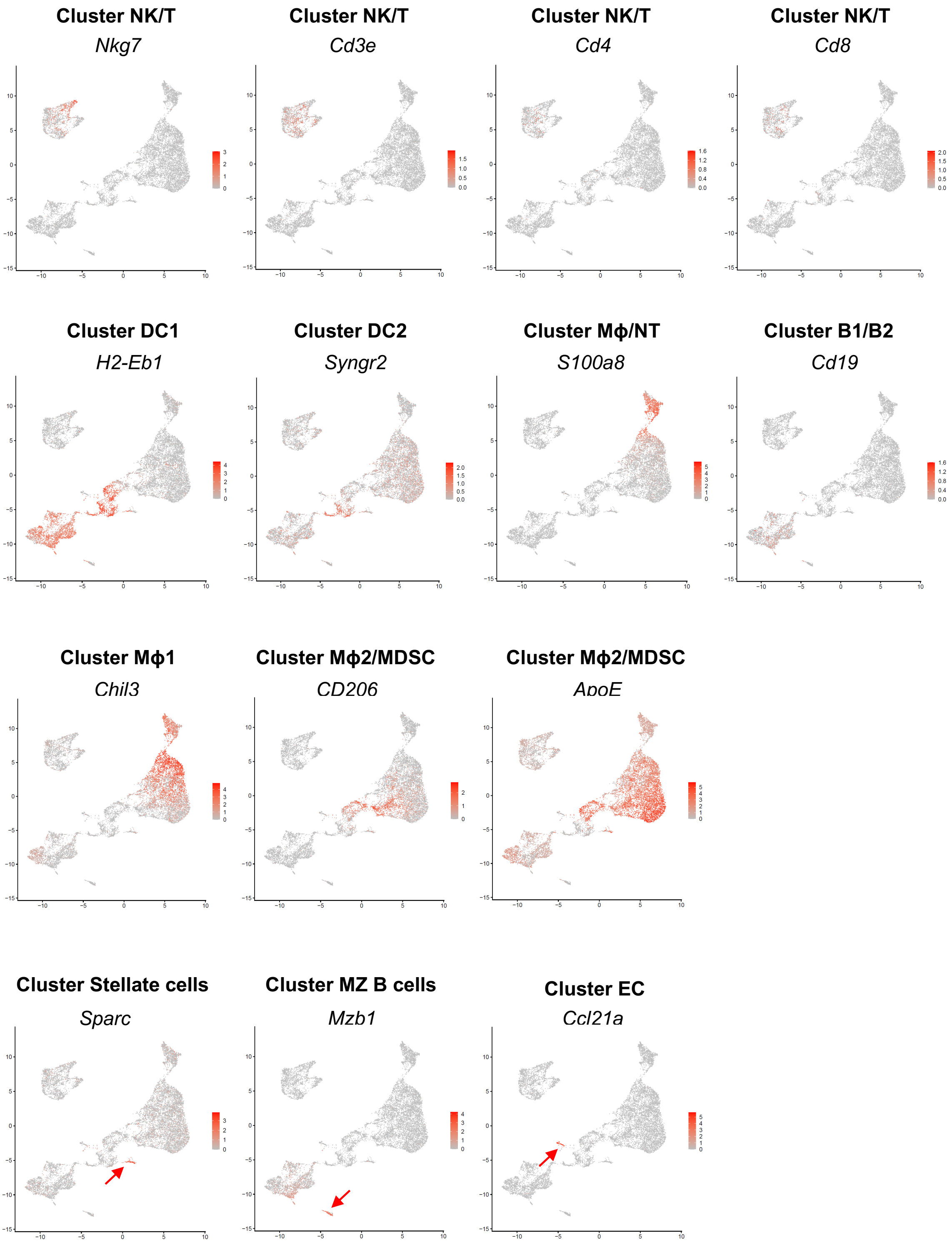

Fig. S1. UMAP showing the expression level of representative markers for each cell type. A. Top markers of each cluster are represented in UMAPs plots. Mϕ = macrophages, NT = neutrophils, DC = dendritic cells, NK = natural killer cells, T = T cells, B = B cells, EC = endothelial cells and MZ = marginal zone.

Development • Supplementary information

Figure S2

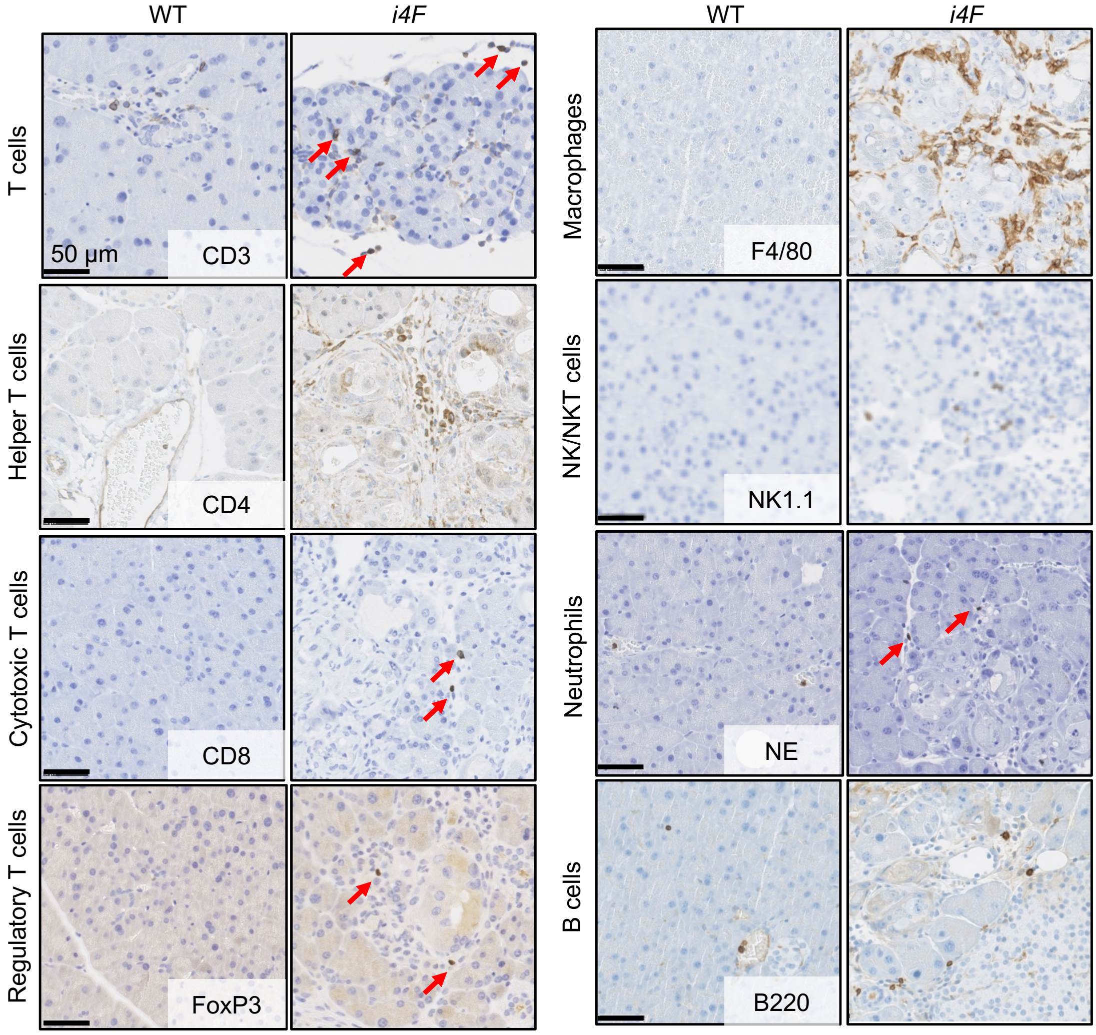

Fig. S2. Immune cells infiltrate partially reprogrammed pancreas. Representative images of partially reprogrammed pancreas at day 7 stained for the indicated immune cell markers (*n*=4): CD3 (T cells), CD4 (helper T cells), CD8 (cytotoxic T cells), FoxP3 (regulatory T cells), F4/80 (macrophages), NK1.1 (NK/NKT cells), NE (neutrophil elastase, neutrophils) and B220 (B cells).

Development • Supplementary information

Figure S3

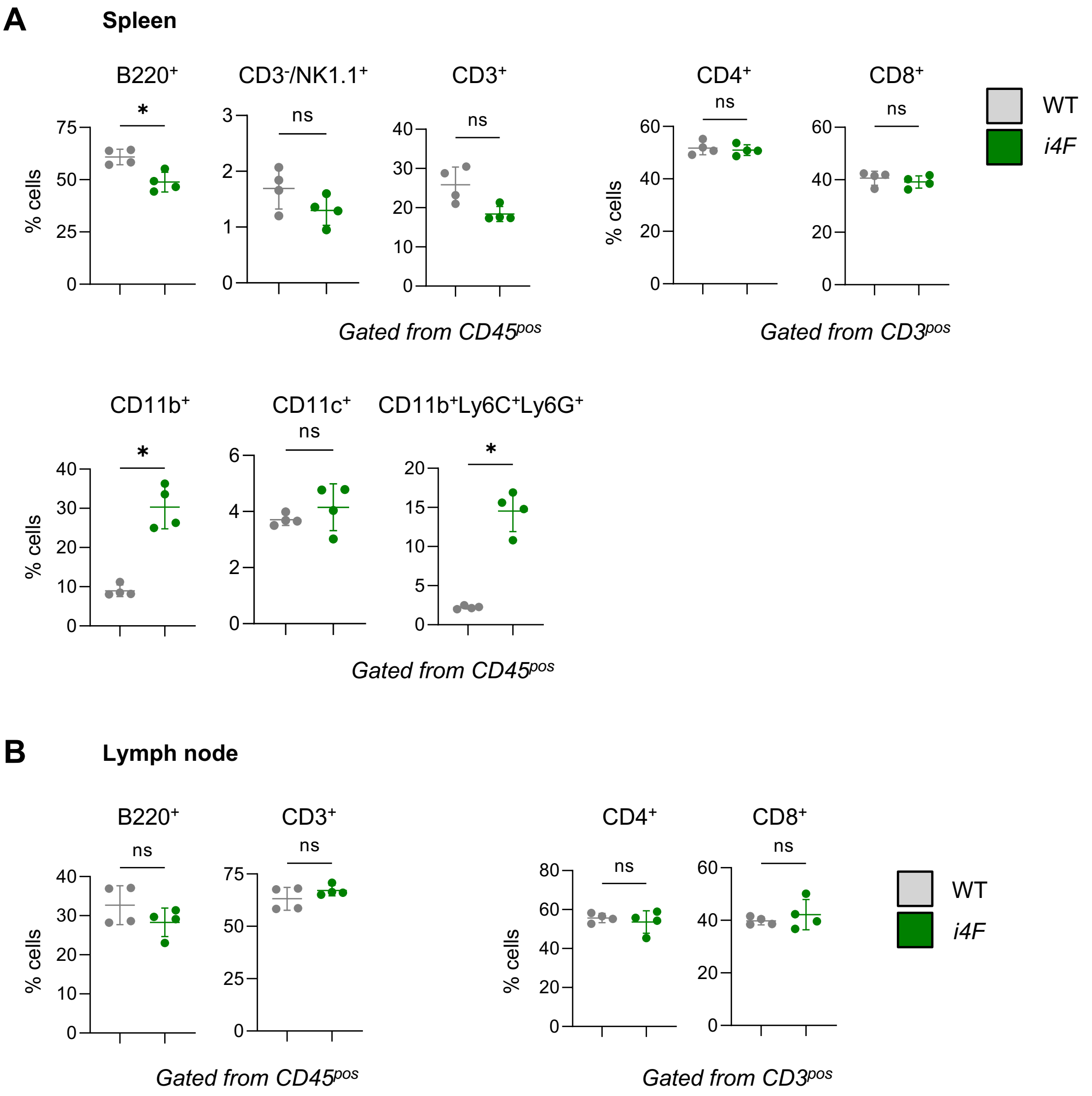

Fig. S3. Gr1+ cells and CD11b+ cells are upregulated in the spleen of *i4F* mice. Spleen and lymph nodes of *i4F* mice treated with doxycycline for 7 days (1 mg/ml) were harvested and most abundant immune populations were analysed by flow cytometry (*n*=4).

Development • Supplementary information

Figure S4

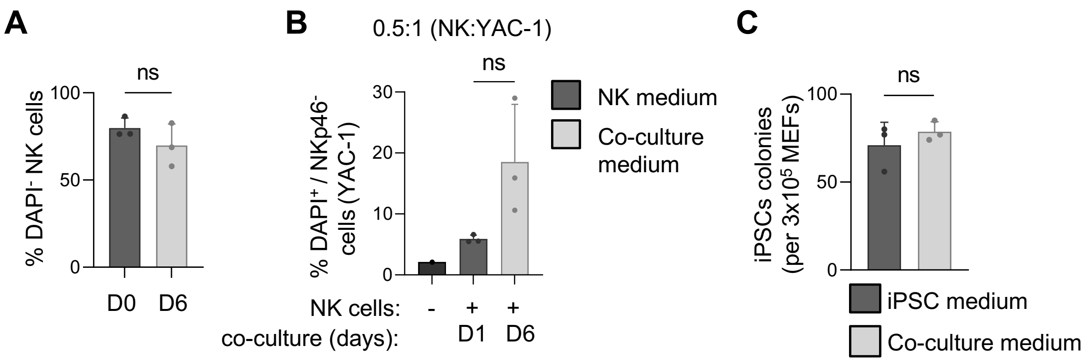

Fig. S4. Co-culture medium maintains NK cell survival and cytotoxicity, and iPSC colony formation. A. Viability of freshly isolated splenic WT NK cells and WT NK cells cultured in co-culture media for 6 days was analyzed. Gated from DAPI$^-$ cells (*n*=3). **B**. Splenic WT NK cells were either primed overnight in NK cell medium (D1) or maintained in co-culture medium for 5 more days (D6). Both conditions were co-cultured with YAC-1 cells for 4 hours to assess their cytotoxic capacity. Cell death was assessed using DAPI and NK cells were excluded using NKp46 antibody (*n*=3). **C**. *i4F* MEFs were reprogrammed *in vitro* with either iPSC medium for 11 days or with iPSC medium replaced by co-culture medium from days 2 to 6 of reprogramming. At day 11, iPSCs colonies were scored by Alkaline Phosphatase staining (*n*=3).

Figure S5

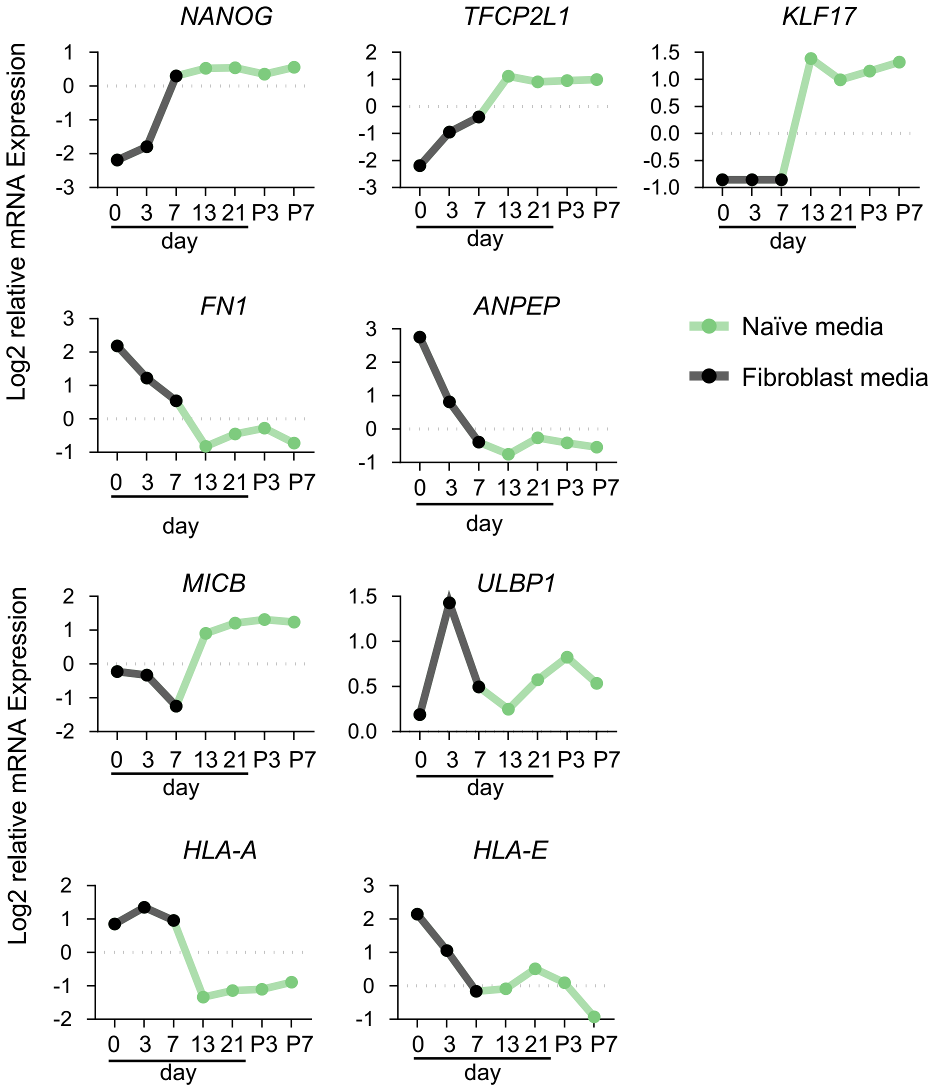

Fig. S5. Human fibroblasts upregulate NK-activating ligand MICB and downregulate MHC-I molecules during reprogramming. Previously published scRNAseq data from human dermal fibroblasts reprogrammed to naïve iPSCs for 21 days (Liu *et al.*, 2020). Cells were transduced with the transcription factors *OCT4*, *KLF4*, *SOX2* and *MYC*, and cultured in fibroblast medium for 7 days. Medium was changed to naïve medium at day 8 and cells were cultured until day 21. Relative mRNA expression of marker genes associated with pluripotency (*NANOG*, *TFCP2L1* and *KLF17*), human fibroblasts (*FN1* and *ANPEP*), NK-activating ligands (*MICB* and *ULBP1*) and MHC-I molecules (*HLA-A* and *HLA-E*) are represented at days 0, 3, 7, 13 and 21, and passages (P) 3 and 7.

Figure S6

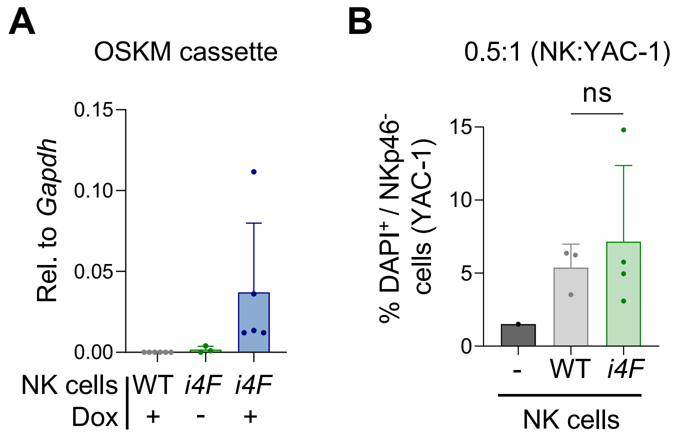

Fig. S6. NK cells maintain their cytotoxic activity upon OSKM induction. A. Spleens were harvested and NK cells were isolated on day 7 post-doxycycline (dox) initiation. Expression of the OSKM transgene was assessed by RT-qPCR (*n*=5 for WT+ dox and *i4F* + dox groups, and *n*=3 for *i4F* group). **B**. Percentage of DAPI$^+$ YAC-1 cells lysed by WT or *i4F* NK cells. Mice were reprogrammed *in vivo* for 5 days and NK cells were isolated from the spleen. NK cells were primed *in vitro* with IL-2 and IL-15 in NK media for 6 days in the presence of doxycycline (1 µg/ml). A cytotoxic assay using YAC-1 as target cells was done after 4 hours of co-culture. Cell death was assessed using DAPI and NK cells were excluded using NKp46 antibody (*n*=3 for WT and *n*=4 for *i4F* group).

Figure S7

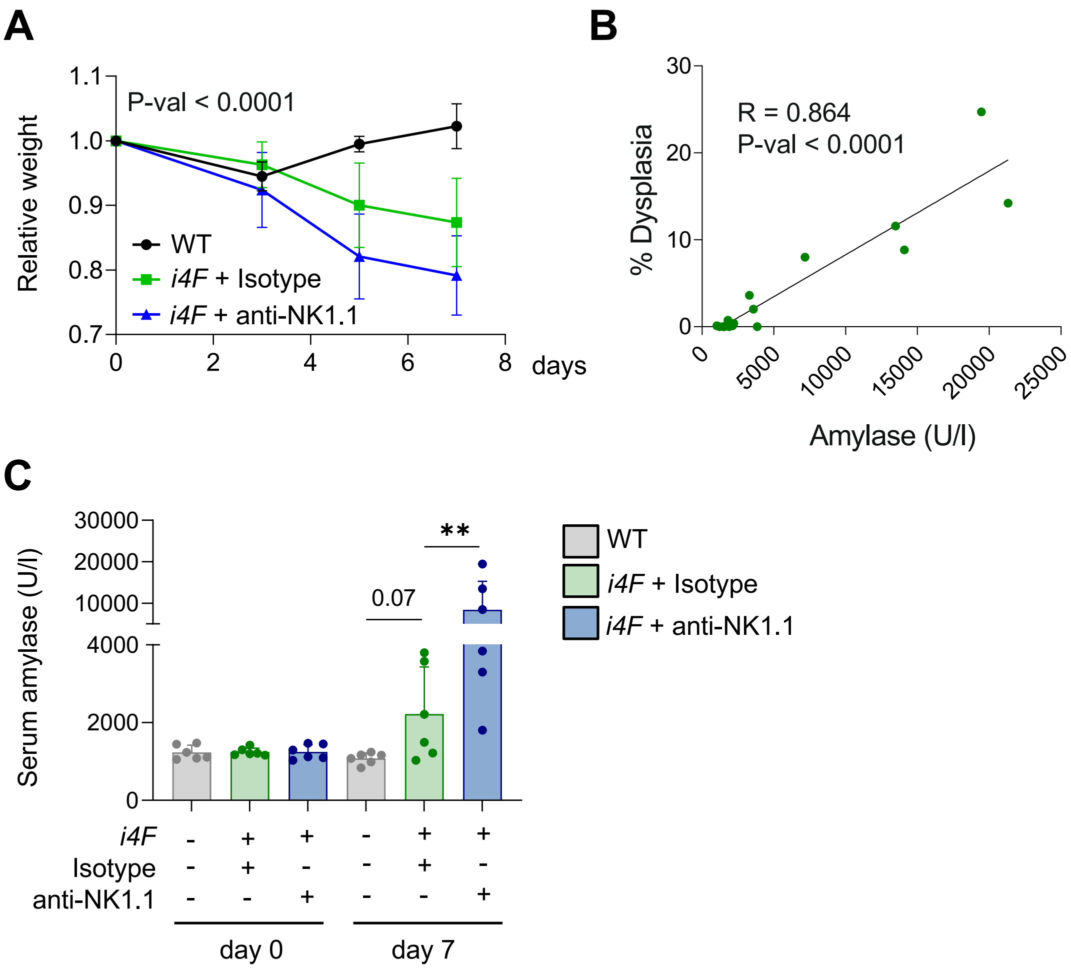

Fig. S7. Reprogrammed *i4F* mice treated with anti-NK1.1 lose more weight and have higher levels of amylase in serum than *i4F* mice treated with isotype control antibody. A. Relative weight loss of mice treated with doxycycline and anti-NK1.1 or isotype control antibodies for 7 days (n = 6). **B**. Correlation between amylase in serum and percentage of dysplasia in pancreas of *i4F* mice at day 7 of reprogramming (n=12). **C**. Amylase levels in serum at days 0 and 7 from reprogrammed mice treated with anti-NK1.1 or isotype control antibodies (n = 6). Graphs represent mean ± SD; **p<0.01 and statistical significance was evaluated using simple lineal regression (**A** and **B**) and two-tailed Student's t-test (**C**).

Table S1. List of primers used for mRNA expression analyses.

Name	Forward	Reverse
E2A-cMyc	GGCTGGAGATGTTGAGAGCAA	AAAGGAAATCCAGTGGCGC
Rae1	ACCCGAATGCAGACAGGAAGTTGA	GGACCTTGAGGTTGATCTTGGCTT
Mult1	CAATGTCTCTGTCCT CGGAA	CTGAACACGTCTCAGGCACT
Icam1	CTGTTTGAGCTGAGCGAGAT	AGGGTGAGGTCCTTGCCTAC
Cd155	CAACTGGTATGTTGGCCTCA	ATTGGTGACTTCGCACACAA
Gapdh	AGGTCGGTGTGAACGGATTTG	TGTAGACCATGTAGTTGAGGTCA

Table S2. List of fluorescent antibodies used for cytometry analyses.

Antibody	Source	Identifier	Dilution
B220 PE	Biolegend	103207	1:200
CD107a BV711	Biolegend	564348	1:100
CD11b A488	BioLegend	101219	1:200
CD11b BV786	BD Biosciences	740861	1:200
CD11b PECy7	eBioscience	25-0112-82	1:300
CD11b PerCPCy5.5	eBioscience	45-0112-82	1:400
CD11c PE-Vio 770	Milteny Biotec	130-110-840	1:200
CD155 PE-Cy7	Biolegend	131512	1:200
CD19-B220 APC-eF780	eBioscience	47-0452-80	1:200
CD3 AF700	eBioscience	56-0032-82	1:100
CD3 APC	eBiosciences	17-0032-80	1:300
CD3 FITC	Biolegend	100203	1:100
CD3ϵ PerCPCy5.5	Biolegend	100327	1:100
CD4 APC-eFluor780	eBioscience	47-0041-80	1:200
CD4 PE EF610	eBioscience	61-0042-80	1:400
CD45 BV605	Biolegend	103139	1:400
CD45 PE	BD Biosciences	553081	1:400
CD45 PerCP	BioLegend	103130	1:200
CD54/ICAM1 FITC	eBioscience	11-0541-82	1:200
CD8 BV786	BD Biosciences	563332	1:400
CD8 FITC	eBioscience	11-0081-82	1:400
F4/80 AF647	BioLegend	123122	1:100
F4/80 APC	BioLegend	123115	1:100
H60 APC	Milteny Biotec	130-108-821	1:100
Ly49CI BV421	BD Biosciences	744027	1:100
Ly6C FITC	BD Biosciences	561085	1:300
Ly6G PE	BD Biosciences	561104	1:300
NK1.1 APCCy7	BioLegend	108723	1:400
NK1.1 BV711	Biolegend	108745	1:200
NKG2A/CD159A PECy7	Biolegend	142809	1:100
NKG2D/CD314 PE	Biolegend	130207	1:100
NKp46-APC/Fire^{TM} 750	BD Biosciences	137631	1:50
Rae1 Pan FITC	Milteny Biotec	130-111-468	1:50
ULBP1/MULT1 PE	R&D Systems	FAB2588P	1:100

Development • Supplementary information
