# Supplementary material for: Natural killer cells act as an extrinsic barrier for in vivo reprogramming
Source: Development. 2022 Apr 22;149(8):dev200361. doi: 10.1242/dev.200361 (PMC9124575; doi:10.1242/dev.200361)
Supplement: Reviewer comments [file develop-149-200361_review_history.pdf]

Natural killer cells act as an extrinsic barrier for *in vivo* reprogramming

Elena Melendez, Dafni Chondronasiou, Lluc Mosteiro, Jaime Martínez de Villareal, Marcos Fernández-Alfara, Cian J. Lynch, Dirk Grimm, Francisco X Real, Jose Alcami, Núria Climent, Federico Pietrocola and Manuel Serrano
DOI: 10.1242/dev.200361

Editor: Florent Ginhoux

Review timeline

Original submission:	15 November 2021
Editorial decision:	23 December 2021
First revision received:	16 February 2022
Accepted:	21 March 2022

Original submission

First decision letter

MS ID#: DEVELOP/2021/200361

MS TITLE: Natural killer cells are an extrinsic barrier for in vivo reprogramming

AUTHORS: Manuel Serrano, Elena Melendez, Dafni Chondronasiou, Lluc Mosteiro, Jaime Martinez de Villareal, Dirk Grimm, Francisco X Real, Jose Alcami, Nuria Climent, and Federico Pietrocola

I have now received all the referees' reports on the above manuscript, and have reached a decision. The referees' comments are appended below, or you can access them online: please go to BenchPress and click on the 'Manuscripts with Decisions' queue in the Author Area.

As you will see, the referees express considerable interest in your work, but have some significant criticisms and recommend a substantial revision of your manuscript before we can consider publication. If you are able to revise the manuscript along the lines suggested, which may involve further experiments, I will be happy receive a revised version of the manuscript. Your revised paper will be re-reviewed by one or more of the original referees, and acceptance of your manuscript will depend on your addressing satisfactorily the reviewers' major concerns. Please also note that Development will normally permit only one round of major revision.

We are aware that you may be experiencing disruption to the normal running of your lab that make experimental revisions challenging. If it would be helpful, we encourage you to contact us to discuss your revision in greater detail. Please send us a point-by-point response indicating where you are able to address concerns raised (either experimentally or by changes to the text) and where you will not be able to do so within the normal timeframe of a revision. We will then provide further guidance. Please also note that we are happy to extend revision timeframes as necessary.

Please attend to all of the reviewers' comments and ensure that you clearly highlight all changes made in the revised manuscript. Please avoid using 'Tracked changes' in Word files as these are lost in PDF conversion. I should be grateful if you would also provide a point-by-point response detailing how you have dealt with the points raised by the reviewers in the 'Response to Reviewers' box. If you do not agree with any of their criticisms or suggestions please explain clearly why this is so.

© 2022. Published by The Company of Biologists under the terms of the Creative Commons Attribution License (https://creativecommons.org/licenses/by/4.0/).

Reviewer 1

Advance summary and potential significance to field

Mendelez et al. examine anti-stem cell differentiation NK cells responses after induction of in vivo pluripotency in mouse models. Their results propose that NK cells limit in vivo pluripotency by eliminating cells under stemness plasticity. This manuscript is well-written, and the study brings important information that advances the current knowledge regarding the biology of NK cell immunosurveillance of stem cells.

Comments for the author

Some aspects can be addressed and considered by the authors for improvement before its resubmitted again. The following comments and modifications are offered:
-Figure 1: It is unclear what is the Figure 1A showing. Are these events gated from leukocyte (CD45+) populations, or in total pancreas homogenates? No enough detail is provided in Fig Legend or methods.
-Figure 2C and 3A seems highly relevant, and a protein expression validation should strength the data. Sometimes RNA expression do not translate to protein expression and this is relevant biology to learn. Authors are encouraged to perform MFI comparison by FACS analysis. In FACS analysis, authors are also encouraged to use NKp46 as a NK cell stain, as it is a more reliable staining than NK1.1.
-Figure 4A. The NKg2A/E/C+ cell data is confusing and might be non NK cell-related. Again, NKp46 stain would be encouraged here, specially as this marker would not be conflicted with the anti-NK1.1 depletion antibody.
- In the sentence "Depletion of NK1.1+ cells allows the survival of highly plastic pancreatic cells". This paragraph cite Figure 6, but I believe authors wanted to cite Figure 7 instead. Please double check and correct for accuracy.
-Referencing. Occasionally low profile and/or old reviews (e.g. Abel et al 2018, Long et al 2013, Quatrini et al 2015, Molfeta et al 2016, Martinet 2015 etc) are cited to describe biological facts in NK and stem cells. Although this is not wrong, this reviewer would encourage the authors to replace these by the original publications that cite the facts they are describing, for correctly referencing the research paper describing it.
- Conclusion. The last paragraph (I suppose it is the conclusion statement) seems vague, and could be more accurate for the NK/Stem immunosurveillance context.
- Reagents cited in method. Seems to be missing the country/state in brands cited. Please double check and describe reagents accurately.
- Method of anti-NK1.1 depletion. It is unclear if this antibody was administered in d-1, 3 and 5 (like others) and then weekly?
- Methods of "Co-culture experiments". NK cells do not survive overnight without IL-2 or IL-15. They also notoriously dislike to be cultured in DMEN. If these co-cultures are performed in day 2 to 6 in DMEN and without IL-2 or IL-15 (e.g. 10ng/mL), the NK cells would just not be alive early already in the culture). It is unclear if the media use NK cell surviving factors (although this could potentially affect the stem cells themselves). For accuracy, a shorter killing kinetics (e.g. Calcein Release for 4h) would suffice to show NK cell killing independently of which media or supplementation of cytokines are present. Authors are encouraged to reconsider conventional NK cell killing (e.g. 51Cr release or Calcein Release) as a method. This would make this data easier and more reproducible.

Reviewer 2

Advance summary and potential significance to field

See below.

Comments for the author

The manuscript by Melendez and colleagues reports the role of natural killer (NK) cells during in vivo reprogramming. The authors observed that during the process of reprogramming, cells express NK activating ligands, which are recognized and killed by NK cells.

© 2022. Published by The Company of Biologists under the terms of the Creative Commons Attribution License (https://creativecommons.org/licenses/by/4.0/).

Moreover, in vivo transient depletion of NK cells improved reprogramming efficiency. Based on these results, the authors conclude that NK cells act as a barrier during in vivo reprogramming.
Comments on the manuscript:
The authors conclude that NK cells are a barrier for in vivo reprogramming as they limit the emergence of dysplastic cells with Nanog expression. First, why do we need complete reprogramming of cells in vivo? Can the authors provide a few examples? Also, under what contexts/disease conditions complete reprogramming of cells in vivo is needed? If the authors believe there are such examples, they should elaborate on them
The concept of partial reprogramming by expressing Yamanaka factors for short periods has been recently demonstrated in different in vivo and in vitro settings.
How do the current observations affect partial reprogramming? Especially, the expression of NK activating ligands are not induced until day-3 and can only be detected starting at day-3 (in vitro) and much later in the pancreas (in vivo).
Does this mean the role of NK cells is only on cells that have passed the early reprogramming state and will not affect partial reprogramming?
In Figure 6, I failed to understand the logic behind this experiment. Control mice grew teratomas and died, suggesting that if NK cells are suppressed, the mice die more quickly because the growth of cancer cells cannot be suppressed. These results do not suggest that NK cells are interfering with the liver regeneration process mediated by in vivo reprogramming. Page 11, Line 343, the authors describe that “These results put forward the hypothesis that monoclonal antibodies targeting specific immune subsets can be deployed to enhance the pro-regeneration potential of reprogramming”. The authors should consider the possibility of inflammation induction by NK cells via in vivo reprogramming.
The authors used the mouse as a model for both in vitro and in vivo experiments.
It will be interesting to analyze the in vivo reprogramming effects of mature immune cells in secondary immune tissues such as the spleen, lymph nodes, and Peyer's patches.
Is the mechanism of NK cells similar in human cells? Did the authors try similar experiments using human cells?
The authors need to describe in detail the impact of in vivo reprogramming on the function of the pancreas. Did the function of the pancreas improve with the infiltration of the immune cells or the opposite?
As the author indicate, 4F reprogramming generally downregulates the presentation of self-antigens by MHC-I, so it is easy to imagine that NK cells recognize them as non-self cells and try to eliminate them. Does the in vivo reprogramming affect and down-regulate the MHC-I expression level in other different organs?
In Figure 1B, the authors need to present a more detailed analysis of immune cell fractions. NK/T should be divided into NK and T cells, and the data of CD4 and CD8 T cell fraction be shown.
In Figure 1B, it seems that there is a significant increase in M1 and M2 macrophages rather than an increase in NK cells. Is this an immune response related to tissue repair or to inflammation? Also, the authors need to describe in detail whether this is the result of reprogramming of non-immune tissue or immune cells.
The authors have previously shown that Yamanaka factors expression in vivo lead to teratoma formation even in the presence of NK cells. Is it because the NK cells have some limitations to attack the cells or do they get exhausted when large populations of cells are undergoing reprogramming simultaneously?
In the liver teratoma experiment, have the authors tried the assay without Myc?

First revision

Author response to reviewers' comments

[AUTHORS] We thank the reviewers for their time and insightful comments. As explained below, we have addressed all their comments and we believe the paper has been further improved.

Reviewer 1:
Melendez et al. examine anti-stem cell differentiation NK cells responses after induction of in vivo pluripotency in mouse models. Their results propose that NK cells limit in vivo pluripotency by eliminating cells under stemness plasticity. This manuscript is well-written, and the study brings

© 2022. Published by The Company of Biologists under the terms of the Creative Commons Attribution License (https://creativecommons.org/licenses/by/4.0/).

important information that advances the current knowledge regarding the biology of NK cell immunosurveillance of stem cells.

[AUTHORS] We truly appreciate this summary of our work and the positive general evaluation.

Some aspects can be addressed and considered by the authors for improvement before its resubmitted again. The following comments and modifications are offered:
-Figure 1: It is unclear what is the Figure 1A showing. Are these events gated from leukocyte (CD45+) populations, or in total pancreas homogenates? No enough detail is provided in Fig Legend or methods.

[AUTHORS] We apologize for not having explained the UMAPs of the scRNAseq in detail. Sequence data in scRNAseq is generally sparse (only a fraction of cells expressing a given marker are actually detected) and for this reason cluster annotation is not based on a single marker, but on a group of markers. In particular, clusters were annotated using the most significant markers of each cluster and the function “FindAllMarkers” of Seurat (v3). This is mentioned now in the legend of Fig. 1 and in Methods (**see revised legend for Figure 1, lines 449-450 of materials and methods and lines 94-95 of results**).

-Figure 2C and 3A seems highly relevant, and a protein expression validation should strength the data. Sometimes RNA expression do not translate to protein expression and this is relevant biology to learn. Authors are encouraged to perform MFI comparison by FACS analysis. In FACS analysis, authors are also encouraged to use NKp46 as a NK cell stain, as it is a more reliable staining than NK1.1.

[AUTHORS] Thanks for mentioning this. We have examined the expression of NK-activating ligands by FACS, both *in vitro* (*i.e.* reprogramming fibroblasts) and *in vivo* (*i.e.* reprogramming pancreas). To our satisfaction, we confirmed by FACS the upregulation of NK-activating ligands (**see new Figures 2D and 3A, and lines 143-148 and 181-186**).

Regarding the use of NKp46 for FACS analyses, please, see next point.

-Figure 4A. The NKg2A/E/C+ cell data is confusing and might be non-NK cell-related. Again, NKp46 stain would be encouraged here, especially as this marker would not be conflicted with the anti-NK1.1 depletion antibody.

[AUTHORS] As suggested by the reviewer, we have performed FACS of NKp46 to assess the depletion of NK cells with anti-NK1.1 (**see new Figure 4B and line 226**).

- In the sentence "Depletion of NK1.1+ cells allows the survival of highly plastic pancreatic cells". This paragraph cite Figure 6, but I believe authors wanted to cite Figure 7 instead. Please double check and correct for accuracy.

[AUTHORS] Thanks for spotting this. Corrected.

-Referencing. Occasionally low profile and/or old reviews (e.g. Abel et al 2018, Long et al 2013, Quatrini et al 2015, Molfeta et al 2016, Martinet 2015 etc) are cited to describe biological facts in NK and stem cells. Although this is not wrong, this reviewer would encourage the authors to replace these by the original publications that cite the facts they are describing, for correctly referencing the research paper describing it.

[AUTHORS] We have revised the references as suggested by the reviewer.

- Conclusion. The last paragraph (I suppose it is the conclusion statement) seems vague, and could be more accurate for the NK/Stem immunosurveillance context.

[AUTHORS] We have modified the last paragraph to make it more accurate. The new paragraph is copied here (**see lines 361-364**).

© 2022. Published by The Company of Biologists under the terms of the Creative Commons Attribution License (https://creativecommons.org/licenses/by/4.0/).

“Cellular dedifferentiation is emerging as a general process during tissue repair and regeneration (Yao and Wang, 2020). The insights obtained in our study, which also involve in vivo dedifferentiation, could be applicable to pathophysiological settings of tissue repair that involve inflammatory cells and, in particular, NK cells.”

- Reagents cited in method. Seems to be missing the country/state in brands cited. Please double check and describe reagents accurately.

[AUTHORS] The Methods section has been revised adding the requested information. We have mentioned the country/state of the product on the first mention.

- Method of anti-NK1.1 depletion. It is unclear if this antibody was administered in d-1, 3 and 5 (like others) and then weekly?

[AUTHORS] As correctly guessed by the reviewer, the administration of NK1.1 in the experiment shown in Figure 6 was performed at days -1, 3 and 5 during the first week, and then once a week until the end of the experiment. This is now clearly stated in Materials and Methods (**see lines 420-421**) and in the legend to Figure 6.

Methods of "Co-culture experiments". NK cells do not survive overnight without IL-2 or IL-15. They also notoriously dislike to be cultured in DMEN. If these co-cultures are performed in day 2 to 6 in DMEN and without IL-2 or IL-15 (e.g. 10ng/mL), the NK cells would just not be alive early already in the culture). It is unclear if the media use NK cell surviving factors (although this could potentially affect the stem cells themselves). For accuracy, a shorter killing kinetics (e.g. Calcein Release for 4h) would suffice to show NK cell killing independently of which media or supplementation of cytokines are present. Authors are encouraged to reconsider conventional NK cell killing (e.g. 51Cr release or Calcein Release) as a method. This would make this data easier and more reproducible.

[AUTHORS] Apologies for overlooking this important detail. The “co-culture medium” that we have used consists in the standard reprogramming medium (“iPSC medium”) supplemented with factors known to be important for NK cells. Namely, IL-2 and IL-15, as well as, HEPES, pyruvate, glutamine, and FBS. The composition of this “co-culture medium” is now clearly explained in Materials and Methods (**see lines 484-489**).

The questions of the reviewer are well taken and have been addressed experimentally:
- Is the long-term viability of NK cells compromised by the “co-culture medium”?: For this, we have measured the viability (DAPI-) of freshly isolated NK cells and the viability of the same cells after 6 days in “co-culture medium”, and we did not observe significant differences. **See new Figure S4A**.
- Is the killing activity of NK cells compromised by the long-term incubation in “co-culture medium”?: We have observed that NK cells incubated for 6 days in “co-culture medium” are as efficient as NK cells incubated for 1 day in standard NK medium in killing YAC-1 cells (4h incubation). **See new Figure S4B**.
- Is reprogramming affected by the “co-culture medium” compared to the standard “iPSC medium?: For this we have performed in parallel reprogramming assays using *i4F* MEFs (in triplicates) and we have obtained the similar numbers of iPSC colonies. **See new Figure S4C**.

Reviewer 2:
The manuscript by Melendez and colleagues reports the role of natural killer (NK) cells during in vivo reprogramming. The authors observed that during the process of reprogramming, cells express NK activating ligands, which are recognized and killed by NK cells. Moreover, in vivo transient depletion of NK cells improved reprogramming efficiency. Based on these results, the authors conclude that NK cells act as a barrier during in vivo reprogramming.

[AUTHORS] We thank the reviewer for this accurate summary of our work.

The authors conclude that NK cells are a barrier for in vivo reprogramming as they limit the emergence of dysplastic cells with Nanog expression. First, why do we need complete reprogramming of cells in vivo? Can the authors provide a few examples? Also, under what

© 2022. Published by The Company of Biologists under the terms of the Creative Commons Attribution License (https://creativecommons.org/licenses/by/4.0/).

contexts/disease conditions complete reprogramming of cells in vivo is needed? If the authors believe there are such examples, they should elaborate on them. The concept of partial reprogramming by expressing Yamanaka factors for short periods has been recently demonstrated in different in vivo and in vitro settings. How do the current observations affect partial reprogramming? Especially, the expression of NK activating ligands are not induced until day-3 and can only be detected starting at day-3 (in vitro) and much later in the pancreas (in vivo). Does this mean the role of NK cells is only on cells that have passed the early reprogramming state and will not affect partial reprogramming?

[AUTHORS] We realize that we have done a poor job at explaining that the *in vivo* reprogramming that we study corresponds indeed to “partial reprogramming”. “Partial reprogramming” is defined by a stage in which cells have lost their identity but have not acquired yet the pluripotent identity. This happens *in vitro* approximately between days 3 and 7 (full reprogramming starts from day 10 to 14, when colonies of iPSCs are formed). “Partial reprogramming” *in vivo* produces a characteristic histological alteration of tissue architecture, which is clearly observable at around day 7 in our mouse model. At this time, the amount of $NANOG^+$ cells is extremely low (as indicated in our quantifications). The presence of these rare cells testifies that the tissue is “in the process” of reprogramming (in contrast to other types of tissue remodeling that do not generate $NANOG^+$ cells, for example tissue injury by caerulein). We have amended the text to make clear that we are studying “partial reprogramming” (**see revised lines 5 (Abstract), 55 (Introduction) 126, 237 and 300 (Results)**).

As correctly indicated by the reviewer, the process of “partial reprogramming” has been shown to be beneficial in several settings, mostly related to increase the reparative capacity of old tissues and organs (Ocampo *et al.*, 2016; de Lázaro *et al.*, 2019; Lu *et al.*, 2020; Chen *et al.*, 2021). These evidences are now mentioned in the Introduction (**see revised Introduction**).

In Figure 6, I failed to understand the logic behind this experiment. Control mice grew teratomas and died, suggesting that if NK cells are suppressed, the mice die more quickly because the growth of cancer cells cannot be suppressed. These results do not suggest that NK cells are interfering with the liver regeneration process mediated by in vivo reprogramming.

[AUTHORS] Again, we realize we have not explained the rationale of this experiment properly. As correctly interpreted by the reviewer, suppression of NK cells facilitates the full process of reprogramming until reaching the teratoma stage. Based on our other data in pancreas and also *in vitro*, we interpret that NK cells are acting as a barrier at the stage of “partial reprogramming”. However, we agree with the reviewer that we cannot exclude that NK cells are preventing teratoma formation at later stages. We now mention this caveat in the text (**see lines 270-271 and 276-278**).

Page 11, Line 343, the authors describe that “These results put forward the hypothesis that monoclonal antibodies targeting specific immune subsets can be deployed to enhance the pro-regeneration potential of reprogramming”. The authors should consider the possibility of inflammation induction by NK cells via in vivo reprogramming.

[AUTHORS] We thank the reviewer for mentioning this. The effects of inflammatory cells on regeneration is an interesting possibility that we had not considered and it is now mentioned in the Discussion (**see line 364**).

The authors used the mouse as a model for both in vitro and in vivo experiments. It will be interesting to analyze the in vivo reprogramming effects of mature immune cells in secondary immune tissues such as the spleen, lymph nodes, and Peyer's patches.

[AUTHORS] As suggested by the reviewer, we have examined the most abundant immune cell populations in the spleen and inguinal lymph nodes. The relative abundance of B, CD8 and CD4 cells was modestly affected or not affected in spleen and lymph nodes. We only observed an increase in $CD11b^+$ cells (mostly macrophages) and $Cd11b^+Ly6G^+Ly6C^+$ (mostly myeloid- derived immunosuppressive cells and neutrophils) in the spleen (**see new Figures S3**). We interpret

© 2022. Published by The Company of Biologists under the terms of the Creative Commons Attribution License (https://creativecommons.org/licenses/by/4.0/).

that this is secondary to the inflammatory process present in the pancreas during “partial reprogramming”.

Is the mechanism of NK cells similar in human cells? Did the authors try similar experiments using human cells?

[AUTHORS] To address this interesting question, we have downloaded publicly available transcriptomic data of human fibroblasts at different times in the reprogramming process (Liu *et al.*, 2020) and we have examined the behavior of NK-stimulatory ligands (**see new Figure S5**). Only a subset of NK-stimulatory ligands were present in these analyses. Interestingly, *MICB* was upregulated. Of note, the NK-inhibitory ligands *HLA-A* and *HLA-E* were downregulated, which would also contribute to NK activation.

The authors need to describe in detail the impact of in vivo reprogramming on the function of the pancreas. Did the function of the pancreas improve with the infiltration of the immune cells or the opposite?

[AUTHORS] We have focused on the transformations of the pancreas “during” reprogramming. In order to answer this question, we have used amylase as a marker of pancreatic damage, as previously reported in human (Quinlan, 2014; Rompianesi *et al.*, 2017). During partial reprogramming (7 days), amylase levels increase, indicating inflammation and dysfunction of the pancreas. Thus, we conclude that the function of the pancreas is impaired “during” partial reprogramming, probably due to the dedifferentiation of acinar cells together with the inflammation originated.

A separate question is how is the function of the pancreas “post-reprogramming”, that is, after a period of recovery post-reprogramming. We know that after 1 week of recovery post-reprogramming, the levels of amylase return to normal levels (**see Figure for the reviewer below**).

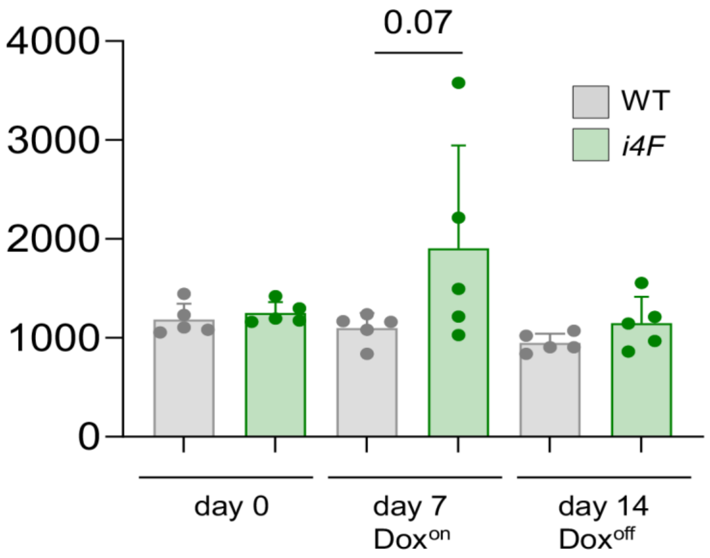

As the author indicate, 4F reprogramming generally downregulates the presentation of self-antigens by MHC-I, so it is easy to imagine that NK cells recognize them as non-self cells and try to eliminate them. Does the in vivo reprogramming affect and down-regulate the MHC-I expression level in other different organs?

[AUTHORS] This is a very interesting point. To answer the reviewer´s question, we analyzed transcriptomic levels of MHC-1a (*H2-Kb*) and MHC-Ib or Qa1b *(H2-T23)* in other organs that undergo partial reprogramming (although to a lower extent than the pancreas), such as the colon, the stomach and the kidney. We observed a general downregulation of MHC-I levels (**see Figure for the reviewer below**).

© 2022. Published by The Company of Biologists under the terms of the Creative Commons Attribution License (https://creativecommons.org/licenses/by/4.0/).

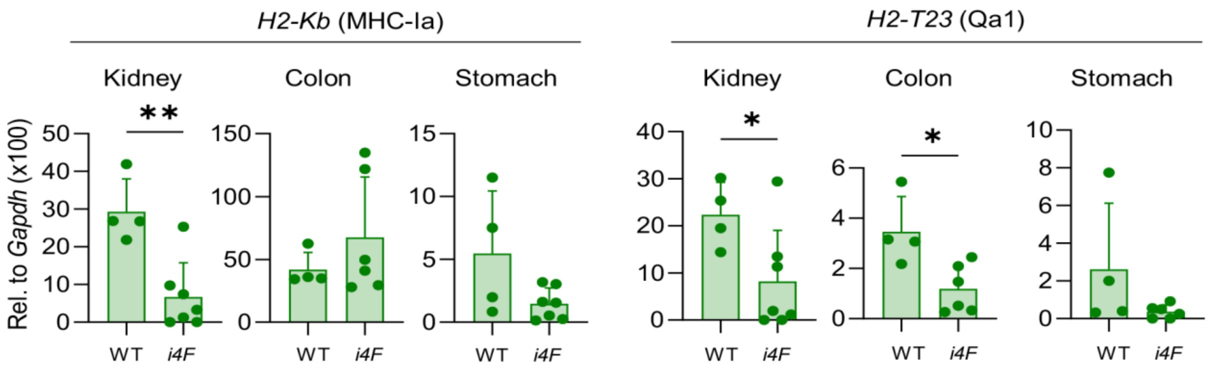

In Figure 1B, the authors need to present a more detailed analysis of immune cell fractions. NK/T should be divided into NK and T cells, and the data of CD4 and CD8 T cell fraction be shown.

[AUTHORS] This figure corresponds to the scRNAseq analyses using 10x Genomics. This method allows to sequence many thousands of cells, as we did to be able to identify sufficient immune cells in the preparation of bulk pancreatic cells. However, this method has the disadvantage of being “shallow” in the sense that only a small subset of mRNAs are captured per cell and this prevents a good separation of all the different cell types. Nonetheless, it is possible to identify unique markers of NK cells (*Nkg7*), CD8 T cells (*Cd8*) and CD4 T cells (*Cd4*). The UMAPs showing the cells positive for each of these markers are now shown in the **revised Figure S1**.

In Figure 1B, it seems that there is a significant increase in M1 and M2 macrophages rather than an increase in NK cells. Is this an immune response related to tissue repair or to inflammation? Also, the authors need to describe in detail whether this is the result of reprogramming of non-immune tissue or immune cells.

[AUTHORS] We agree with the reviewer that the overrepresentation of macrophages during partial reprogramming is considerable. For this reason, we have performed a reprogramming experiment in mice treated with clodronate. Interestingly, despite a profound depletion in macrophages, the extent of partial reprogramming in the pancreas was not affected (**see Figure 5**). This result is mentioned in the text (**see lines 253-255**). Given the lack of effect of macrophage depletion on reprogramming we have not characterized in further detail their recruitment or possible functions. An additional comment in relation to this point is that injury of the exocrine pancreas with caerulein also results in massive recruitment of macrophages (**see Figure below for the reviewer**).

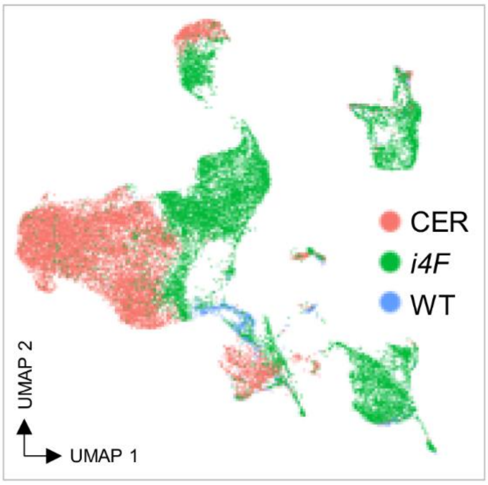

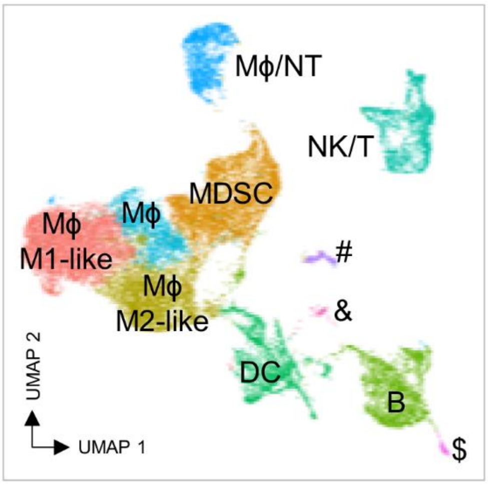

The authors have previously shown that Yamanaka factors expression in vivo lead to teratoma formation even in the presence of NK cells. Is it because the NK cells have some limitations to attack the cells or do they get exhausted when large populations of cells are undergoing reprogramming simultaneously?

© 2022. Published by The Company of Biologists under the terms of the Creative Commons Attribution License (https://creativecommons.org/licenses/by/4.0/).

[AUTHORS] In line with the suggestion of the reviewer, we also think that the amount of cells undergoing reprogramming is so high that it overcomes the killing capacity of NK cells. We have not examined the possible exhausted state of NK cells in advanced reprogramming, although we note that there is upregulation of NKG2A (**see Figure 3D**) and this receptor has been proposed to be associated to NK exhaustion in humans (Ablamunits et al., 2011; Sun et al., 2017) (**see lines 199-204**).

In the liver teratoma experiment, have the authors tried the assay without Myc?

[AUTHORS] These are experiments that were reported in our previous paper using AAVs to induce liver reprogramming and teratomas (Senís *et al.*, 2018). The omission of Myc dramatically reduces the efficiency of reprogramming and teratoma formation: OSKM produces teratomas in 100% of the mice after 6 weeks; OSK produces teratomas in 33% of the mice after 1 year.

References cited:

Ablamunits, V. *et al.* (2011) 'NKG2A is a marker for acquisition of regulatory function by human CD8+ T cells activated with anti-CD3 antibody', *European Journal of Immunology*, 41(7), pp. 1832-1842. doi: 10.1002/eji.201041258.

Chen, Y. *et al.* (2021) 'Reversible reprogramming of cardiomyocytes to a fetal state drives adult heart regeneration in mice', *Science*, 1540(September), pp. 1537–1540.

de Lázaro, I. *et al.* (2019) 'Non-viral, Tumor-free Induction of Transient Cell Reprogramming in Mouse Skeletal Muscle to Enhance Tissue Regeneration', *Molecular Therapy*, 27(1), pp. 59- 75. doi: 10.1016/j.ymthe.2018.10.014.

Liu, X. *et al.* (2020) 'Reprogramming roadmap reveals route to human induced trophoblast stem cells', *Nature*, 586(7827), pp. 101–107. doi: 10.1038/s41586-020-2734-6.

Lu, Y. *et al.* (2020) 'Reprogramming to recover youthful epigenetic information and restore vision', *Nature*, 588(7836), pp. 124–129. doi: 10.1038/s41586-020-2975-4.

Ocampo, A. *et al.* (2016) 'In Vivo Amelioration of Age-Associated Hallmarks by Partial Reprogramming', *Cell*, 167(7), pp. 1719-1733.e12. doi: 10.1016/j.cell.2016.11.052.

Quinlan, J. D. (2014) 'Acute pancreatitis', *American Family Physician*, 90(9), pp. 632-639. doi: 10.5005/jp/books/13118_50.

Rompianesi, G. *et al.* (2017) 'Serum amylase and lipase and urinary trypsinogen and amylase for diagnosis of acute pancreatitis.', *The Cochrane database of systematic reviews*, 4(4), p. CD012010. doi: 10.1002/14651858.CD012010.pub2.

Senís, E. *et al.* (2018) 'AAVvector-mediated in vivo reprogramming into pluripotency', *Nature Communications*, 9(1), pp. 1-14. doi: 10.1038/s41467-018-05059-x.

Sun, C. *et al.* (2017) 'High NKG2A expression contributes to NK cell exhaustion and predicts a poor prognosis of patients with liver cancer', *OncoImmunology*, 6(1), pp. 1–12. doi: 10.1080/2162402X.2016.1264562.

Yao, Y. and Wang, C. (2020) 'Dedifferentiation: inspiration for devising engineering strategies for regenerative medicine.', *NPJ Regenerative medicine*, 5, p. 14. doi: 10.1038/s41536-020- 00099-8.

Second decision letter

MS ID#: DEVELOP/2021/200361

MS TITLE: Natural killer cells act as an extrinsic barrier for in vivo reprogramming

AUTHORS: Elena Melendez, Dafni Chondronasiou, Lluc Mosteiro, Jaime Martinez de Villareal, Marcos Fernandez-Alfara, Cian Lynch, Dirk Grimm, Francisco X Real, Jose Alcami, Nuria Climent, Federico Pietrocola, and Manuel Serrano
ARTICLE TYPE: Research Article

I am happy to tell you that your manuscript has been accepted for publication in Development, pending our standard ethics checks.

© 2022. Published by The Company of Biologists under the terms of the Creative Commons Attribution License (https://creativecommons.org/licenses/by/4.0/).

Reviewer 1

Advance summary and potential significance to field

Authors did a great job during the revision, and the paper was substantially improved.

Comments for the author

Authors did a great job during the revision, and the paper was substantially improved.

© 2022. Published by The Company of Biologists under the terms of the Creative Commons Attribution License (https://creativecommons.org/licenses/by/4.0/).
